# Therapeutic applications of interface-mimicking peptides for targeting the SARS-CoV-2 NSP12-NSP8 RdRp complex

**DOI:** 10.1016/j.ymthe.2025.05.028

**Published:** 2025-05-27

**Authors:** Mark Anthony B. Casel, Jae-Woo Ahn, Hyunjoon Kim, Isaac Choi, Seung-Gyu Jang, Rare Rollon, Ho-Young Ji, Mina Yu, Seong Cheol Min, Min-Suk Song, Young Ki Choi

**Affiliations:** 1 Center for Study of Emerging and Re-emerging Viruses, Korea Virus Research Institute, Institute for Basic Science (IBS), Daejeon 34126, Republic of Korea; 2 College of Medicine and Medical Research Institute, Chungbuk National University, Cheongju 28644, Republic of Korea; 3 Department of Metabiohealth, Sungkyun Convergence Institute, Sungkyunkwan University (SKKU), Suwon 16419, Republic of Korea

**Keywords:** SARS-CoV2, RdRp complex, NSP12-NSP8 interface, antiviral peptides, NSP8-based peptides

## Abstract

Severe acute respiratory syndrome coronavirus 2 (SARS-CoV-2) replication depends on the NSP12-NSP8-NSP7 complex, which plays a critical role in enhancing RNA-dependent RNA polymerase (RdRp) activity. NSP8 is particularly essential, stabilizing the RdRp complex and supporting viral replication across diverse variants. To disrupt this crucial interaction, we designed four NSP8-derived peptides—N8-Pepα, N8-Pepα_cyc, N8-Pepβ, and N8-PepβD—targeting a key hotspot region within the NSP12-NSP8 interface that governs complex stability and processivity. *In vitro* assays demonstrated that these peptides effectively inhibit RdRp activity by disrupting the NSP12-NSP8 interaction, leading to significant reductions in SARS-CoV-2 replication in Vero E6 cells. Notably, intranasal administration of N8-Pepα or N8-Pepα_cyc (25 mg/kg) in Balb/c mice provided robust antiviral protection, alleviating weight loss and reducing mortality following challenge with a mouse-adapted SARS-CoV-2 strain. Both prophylactic and therapeutic treatments significantly lowered viral titers and minimized pathological damage in the nasal turbinates and lungs. These results highlight the NSP12-NSP8 interface as a novel and highly conserved target for antiviral therapy and establish NSP8-derived peptides, particularly N8-Pepα and N8-Pepα_cyc, as promising candidates for inhibiting RdRp complex formation and controlling SARS-CoV-2 replication.

## Graphical abstract

**Figure:**
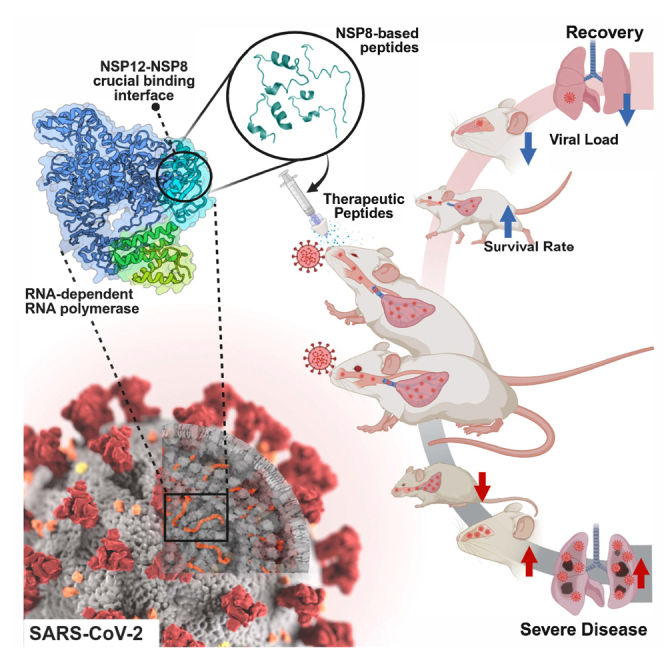

## Teaser

Choi and colleagues designed NSP8-derived peptides (N8-Pepβ, N8-PepβD, N8-Pepα, N8-Pepα_cyc) targeting SARS-CoV-2’s NSP12-NSP8 interface, disrupting RdRp activity. All peptides reduced viral replication *in vitro*; N8-Pepα and N8-Pepα_cyc protected mice, lowering viral titers and disease severity. This conserved interface offers a novel antiviral target, with N8-Pepα_cyc as a promising therapeutic candidate.

## Introduction

Severe acute respiratory syndrome coronavirus 2 (SARS-CoV-2), the causative agent of COVID-19, is a positive-sense single-stranded RNA virus belonging to the *Betacoronavirus* genus.^1^^,^^2^ The polycistronic RNA genome of SARS-CoV-2 encodes multiple structural and non-structural proteins essential for viral RNA synthesis and processing.^3^ Among these, the RNA-dependent RNA polymerase (RdRp) complex, consisting of non-structural proteins NSP12, NSP7, and NSP8, plays a central role in driving efficient viral replication.^2^^,^^4^ Recent studies underscore the critical role of NSP8 in stabilizing the RdRp machinery, where its interaction with NSP12 enhances the processivity and efficiency of RNA synthesis.^5^^,^^6^ This interaction has been implicated in the high replication rates and increased virulence in emerging SARS-CoV-2 variants.^5^ Moreover, the NSP12-NSP8 interface is highly conserved among coronaviruses, making it a potential universal target for antiviral interventions.

While substantial progress has been made in understanding the molecular mechanisms of SARS-CoV-2 replication,^7^^,^^8^^,^^9^^,^^10^ effective therapeutic options remain limited. Currently, among the US Food and Drug Administration (FDA)-approved therapeutics, small-molecule inhibitors such as remdesivir (Veklury), a nucleoside analog that targets the viral RdRp, and nirmatrelvir-ritonavir (Paxlovid), a protease inhibitor targeting the main protease (Mpro), have been widely utilized in clinical settings.^11^^,^^12^^,^^13^ Additionally, molnupiravir, another nucleoside analog, was granted emergency use authorization (EUA) for its ability to introduce lethal mutagenesis into the viral genome, reducing viral replication.^14^ However, the FDA-approved antivirals targeting NSP12, remdesivir and molnupiravir, show only partial efficacy, with variable clinical outcomes and the emergence of resistant variants posing significant challenges.^11^^,^^12^^,^^14^ These limitations highlight the urgent need for innovative therapeutic strategies targeting essential viral replication machinery.

Emerging evidence suggests that disrupting protein-protein interactions within the RdRp complex, specifically targeting the NSP12-NSP8 interface could be a promising therapeutic approach. Recent studies have identified the NSP12 residue P323 as a critical hotspot for this interaction, with the P323L mutation conferring a selective advantage by enhancing RdRp stability and activity.^15^^,^^16^ Notably, this mutation induces conformational changes in a loop (residues 323–327) that strengthen the NSP12-NSP8 binding, making this interface a prime target for therapeutic intervention.

Peptide-based inhibitors offer a compelling strategy for targeting protein-protein interactions.^17^^,^^18^ Peptidyl inhibitors targeting viral entry, replication, and protease activity have demonstrated promising antiviral activity in preclinical studies. Fusion-inhibitory peptides such as HR2P and EK1 disrupt spike-mediated membrane fusion, with the lipopeptide-conjugated EK1C4 showing potent inhibition.^19^^,^^20^ Peptidomimetic compounds like GC376, originally developed for feline coronavirus, have also shown efficacy in inhibiting the SARS-CoV-2 Mpro,^21^ while UAWJ248, a lipopeptide-based inhibitor, offers enhanced stability and Mpro-targeting activity.^22^ Due to their high specificity and adaptability, peptides can be designed to selectively bind and disrupt key interaction interfaces, including those within viral replication machinery.^23^ Leveraging this concept, we designed NSP8-derived peptides to target the identified hotspot region at the NSP12-NSP8 interface.

In this study, we demonstrate that these peptides effectively inhibit RdRp activity and suppress SARS-CoV-2 replication *in vitro*, using Vero E6 cells. *In vivo*, intranasal administration of select peptides in a Balb/c mouse model significantly reduced viral titers in the nasal turbinates and lungs, while providing robust protection against pulmonary damage and mortality following a lethal SARS-CoV-2 challenge. Our findings establish the NSP12-NSP8 interaction as a viable target for antiviral intervention and introduce NSP8-derived peptides as a novel class of therapeutics for combating SARS-CoV-2. This study not only highlights a new approach to addressing the ongoing threat of COVID-19 and its variants but also lays the groundwork for developing broad-spectrum antiviral agents targeting conserved RdRp complexes in related coronaviruses.

## Results

### Design of NSP8-mimicking peptides targeting the NSP12-NSP8 interface

SARS-CoV-2 replication is critically dependent on the RdRp complex, primarily translated from ORF1a and ORF1b.^24^ While NSP12 serves as the catalytic core of the complex, its functional stability and processivity require interactions with the co-factors NSP8 and NSP7, with NSP8 playing a pivotal role in stabilizing the RdRp machinery and enhancing polymerase activity.^25^^,^^26^

**Figure 1:**
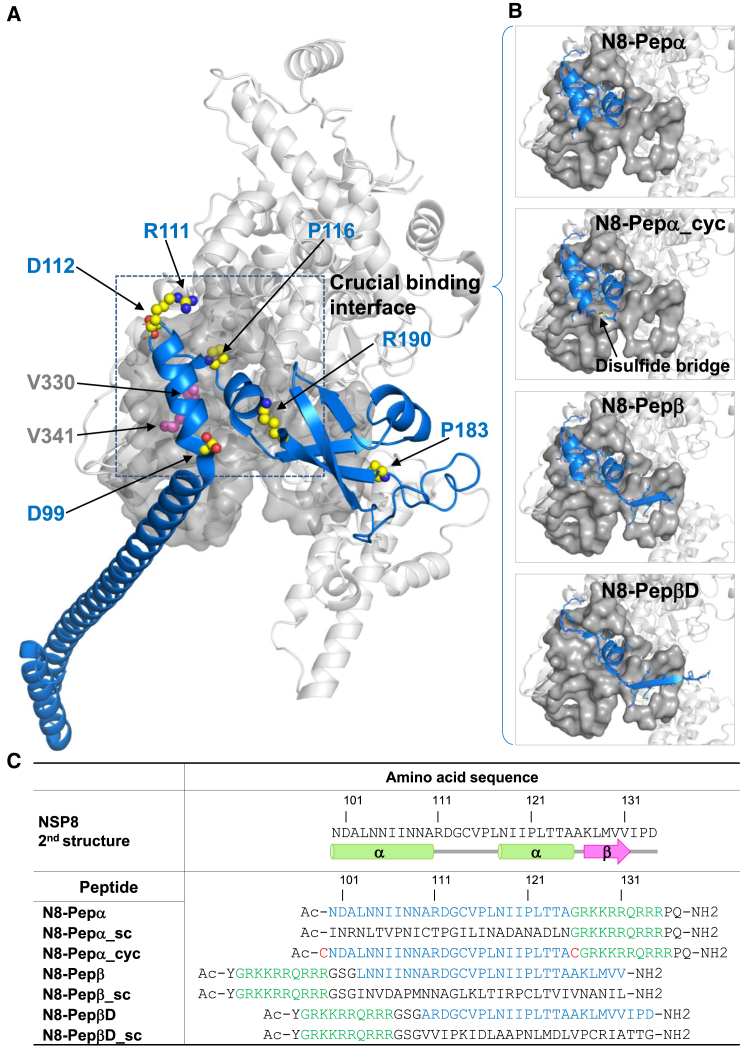
Peptides mimicking for binding to NSP12 (A) Crucial binding site of NSP12-NSP8 interface. The residues associated with RdRp activity are depicted as balls. NSP12, NSP8, and NSP7 are represented as gray, blue, and dark blue illustrations, respectively. The NSP12 interface of NSP12-NSP8 interaction is shown as a semi-transparent dark-gray surface. (B) Designed peptides based on the complex structure. NSP12 and the designed peptide are shown in gray and blue, respectively. N8-Pepα, NSP8 residues 100–125; N8-Pepα_cyc, NSP8 residues 99–126 with C–C bond by C99 and C126; N8-Pepβ, residues 103–131; N8-PepβD, residues 110–134. The NSP8-binding interface on NSP12 is also rendered as a dark gray surface. (C) Peptide sequences of candidate peptides and their scrambled version. Scrambled peptides were generated by shuffling the amino acid sequence of the active peptides while preserving their overall composition and length. The blue letters indicate amino acid sequence of the designed peptide. Green letters represent the TAT peptide, and red “C” represents the cysteine residue introduced for the C–C bond formation.

Given the essential role of NSP8, we hypothesized that disrupting the NSP12-NSP8 interface could inhibit RdRp activity and suppress viral replication. To identify potential target regions, we analyzed the protein-protein interactions within the NSP12-NSP8-NSP7 complex using the cryoelectron microscopy structure (PDB: 6YYT)^27^ and Prot-prot interface analysis from PDBsum.^28^ The results demonstrate the interaction strengths in the order NSP12-NSP8_A > NSP7-NSP8_B > NSP12-NSP7 > NSP8_A-NSP7 > NSP12-NSP8_B (Figures S1A and S1B). Among these, the NSP12-NSP8_A interface exhibited the strongest interaction, supported by a large binding surface (2,388:2,499 Å^2^) and key salt bridges critical for binding stability. Mutational studies further corroborated the importance of specific residues, including D99, R111, D112, P116, and R190 on NSP8 and V330 and V341 on NSP12, which significantly impact polymerase activity.^26^^,^^29^ Notably, D99, R111, D112, and P116 of NSP8 cluster at a crucial binding interface with NSP12 residues V330 and V341, making this region an ideal target for therapeutic intervention (Figure 1A).

Based on these insights, we designed a series of NSP8-mimicking peptides aimed at competitively inhibiting the interaction between NSP8 and NSP12 by binding to their crucial binding interface (Figure 1B). The N8-Pepα peptide encompasses residues 100–125 of NSP8 and includes two α-helices (spanning residues 100–110 and 118–125) containing key interaction residues R111, D112, and P116 that are predicted to interact with V330 and V341 on NSP12. The N8-Pepβ spans residues 103–131, featuring two α helices and an additional β strand (residues 127–131), providing a closer structural resemblance to the full-length NSP8. The N8-PepβD was designed with an extended sequence from residues 110–134, including a single α helix and a β strand, terminating at D135 to enhance solubility while preserving functional activity. Finally, the N8-Pepα_cyc was developed as a cyclized version of N8-Pepα by introducing cysteine residues at D99 and A126 to form a stabilizing disulfide bond, improving peptide stability. For intracellular delivery, all peptides were conjugated with the HIV-trans-activator of transcription (TAT) cell-penetrating peptide (CPP),^30^^,^^31^ either at the N or C terminus. Scrambled versions of each peptide were also synthesized as controls to assess cellular uptake efficiency and specificity during subsequent experiments (Figure 1C).

### Evaluating peptide binding to NSP12 via docking, MST, and SPR analysis

**Figure 2:**
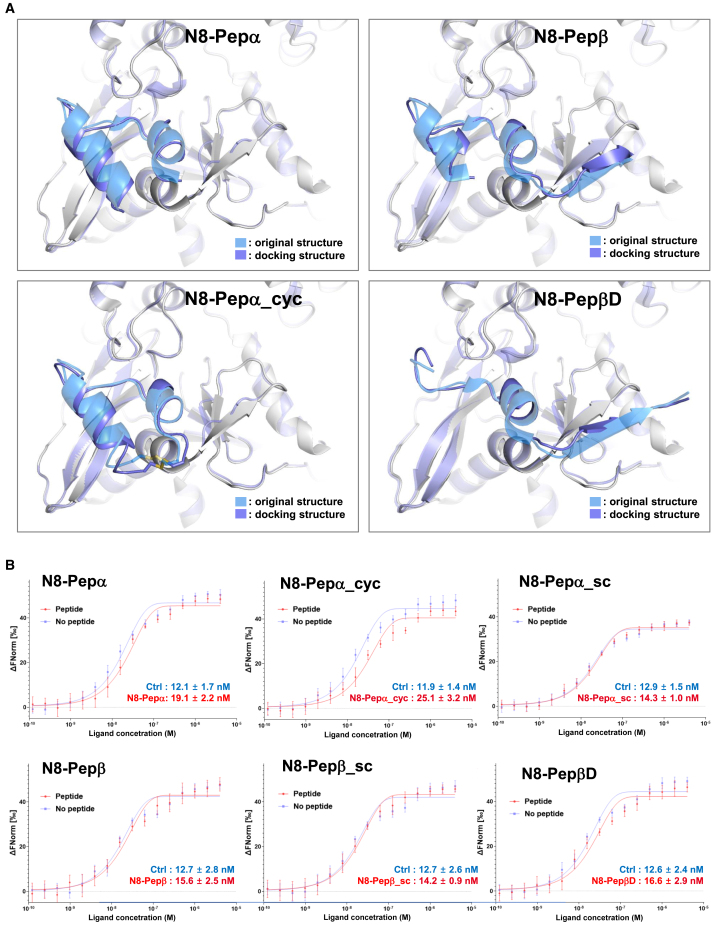
Evaluating inhibitory effect of peptides (A) Superpositions of designed peptides and docking simulations. The original peptide structure, docking structure of peptide, and the NSP12 structure are represented as illustrations colored by sky blue, purple, and gray, respectively. (B) Binding affinity changes were measured by MST. Blue and red lines imply binding affinities (*K*_D_) in the presence and absence of the peptides. The dissociation constant (*K*_D_) was calculated by measuring the change in fluorescence (*ΔFnorm*). The data are expressed as mean ± SD from more than three independent analyses.

To assess the binding affinity of our designed peptides to NSP12 and their structural similarity to native NSP8, docking simulations were performed using HADDOCK (high ambiguity-driven docking) (Table S1). These simulations generated 170, 156, 130, and 177 structures for N8-Pepα, N8-Pepα_cyc, N8-Pepβ, and N8-PepβD, respectively, which were organized into 8, 13, 14, and 8 clusters^32^^,^^33^ representing 85%, 78%, 65%, and 88% of water-refined models. The top clusters for each peptide exhibited HADDOCK scores of −111.9 ± 4.0 for N8-Pepα, −113.2 ± 5.3 for N8-Pepα_cyc, −159.3 ± 3.3 for N8-Pepβ, and −158.6 ± 1.2 for N8-PepβD, indicating strong binding affinities to NSP12. Notably, the highest-ranked docking poses demonstrated high structural similarity to the native binding model of NSP8 (Figure 2A), suggesting that these peptides could effectively compete with NSP8 for NSP12 binding and inhibit complex formation.

To experimentally validate the inhibitory potential of the peptides on NSP12-NSP8-NSP7 complex formation, microscale thermophoresis (MST) was used to measure changes in binding affinities (*K*_D_) of the NSP8-NSP7-RNA ligand complex to NSP12 in the presence of each peptide and its scrambled control (Figure 2B). N8-Pepβ and N8-PepβD produced *K*_D_ values of 15.6 ± 2.5 nM and 16.6 ± 2.9 nM, corresponding to 1.2- and 1.3-fold reductions in binding affinity, respectively. N8-Pepα further reduced the binding affinity to 19.1 ± 2.2 nM, representing a 1.5-fold reduction relative to the control. The most significant inhibitory effect was observed with N8-Pepα_cyc, which yielded a *K*_D_ of 25.1 ± 3.2 nM, indicating a 2.1-fold reduction in binding affinity. Scrambled control peptides, N8-Pepα_sc and N8-Pepβ_sc, showed *K*_D_ values of 14.3 ± 1.0 nM and 14.2 ± 0.9 nM, similar to the unmodified control, confirming that these scrambled peptides did not interfere with complex formation. However, the *K*_D_ for N8-PepβD_sc could not be determined due to peptide aggregation. Collectively, these results identify N8-Pepα_cyc as the most potent inhibitor of NSP12-NSP8-NSP7 complex formation, followed by N8-Pepα, N8-Pepβ, and N8-PepβD, which exhibited moderate inhibitory effects.

To further validate these interactions and quantify direct peptide-NSP12 binding, we performed surface plasmon resonance (SPR) analysis (Figure S2; Table S2). N8-Pepα_cyc exhibited a *K*_*D*_ of 66.5 ± 0.5 nM, with an association rate constant (*k*_*on*_) of 1.57 × 10^4^ M^−1^ s^−1^ and a dissociation rate constant (*k*_*off*_) of 1.04 × 10^−3^ s^−1^. N8-Pepα and N8-Pepβ showed similar affinities, with *K*_*D*_ values of 42.2 ± 4.3 and 43.7 ± 4.8 nM, respectively. Their *k*_*on*_ and *k*_*off*_ values were 1.21 × 10^4^ M^−1^ s^−1^/0.53 × 10^−3^ s^−1^ for N8-Pepα and 1.21 × 10^4^ M^−1^ s^−1^/0.52 × 10^−3^ s^−1^ for N8-Pepβ. While N8-PepβD showed detectable binding, its kinetic parameters were inconsistent and accompanied by a notably low *R*_*max*_, suggesting a weak or unstable interaction. These results confirm that N8-Pepα, N8-Pepα_cyc, and N8-Pepβ bind directly to NSP12 with moderate affinity, while N8-PepβD interacts only weakly.

### Assessing inhibitory efficacy against RdRp activity and cytotoxicity in cells

**Figure 3:**
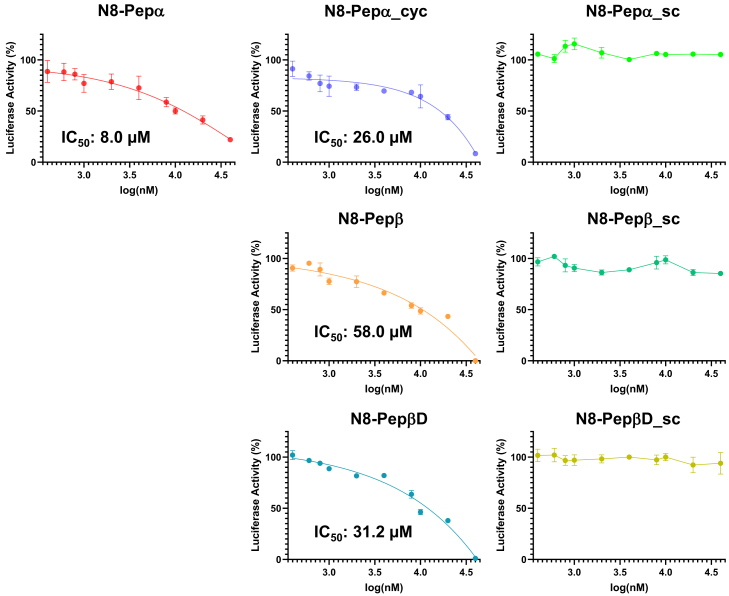
SARS-CoV-2 RdRp activity inhibition by peptides in minigenome assay Dose-response curves illustrate peptide-mediated inhibition of luciferase activity, with IC_50_ values determined for each peptide. The data, representative of at least three independent experiments, are expressed as means.

To evaluate the ability of the NSP8-based peptides to inhibit SARS-CoV-2 RdRp activity, a cell-based SARS-CoV-2 RdRp minigenome assay was performed, as described previously (Figure S3).^5^ This assay demonstrated a dose-dependent reduction in polymerase activity for all peptides, in parallel with the remdesivir, confirming their inhibitory effects on RdRp function (Figure S4). Specifically, the half-maximal inhibitory concentrations (IC_50_s) were approximately 8.0 μM for N8-Pepα, 26.0 μM for N8-Pepα_cyc, 58.0 μM for N8-Pepβ, and 31.2 μM for N8-PepβD (Figure 3). Among these, N8-Pepα exhibited the strongest inhibition, while N8-Pepα_cyc and N8-PepβD showed relatively weaker IC_50_ values, 3–4 times higher than N8-Pepα, with N8-Pepβ having the weakest IC_50_. Negative control peptides showed no inhibitory activity, comparable to the mock-treated control.

Cytotoxicity was assessed using an MTT (3-[4,5-dimethylthiazol-2-yl]-2,5-diphenyl tetrazolium bromide) assay on Vero-E6 cells transfected with SARS-CoV-2 RdRp minigenome plasmids. Peptide concentrations ranged from 0.2 to 40 μM (Figure S5), and cell viability was analyzed following ISO 10993-5 guidelines.^34^ The results indicated no cytotoxic effects for all peptides at concentrations up to 10 μM. At higher concentrations, N8-Pepα and N8-Pepα_cyc maintained cell viability above 80% even at 40 μM, suggesting no cytotoxicity, while N8-Pepβ and N8-PepβD displayed weak cytotoxicity at concentrations between 20 and 40 μM. Importantly, the IC_50_ values for N8-Pepα, N8-Pepα_cyc, and N8-PepβD were within the range of concentrations exhibiting mild or no toxicity, indicating that these peptides are safe at their effective inhibitory doses.

### Inhibitory effects of the NSP8-based peptide on SARS-CoV-2 replication

**Figure 4:**
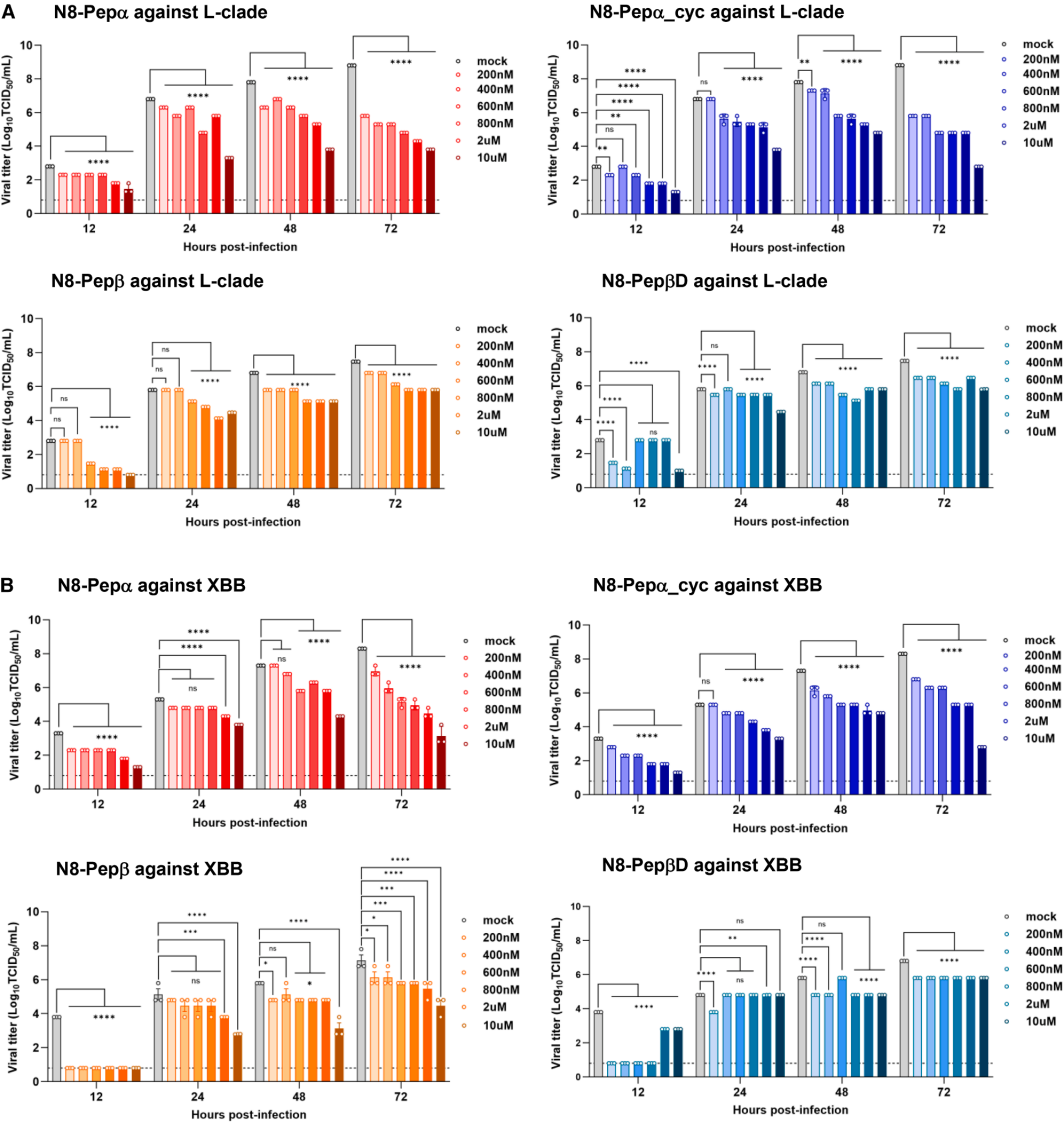
Inhibitory effects of NSP8-based peptide in SARS-CoV-2 replication SARS-CoV-2 infectious virus titers in Vero E6 cells following different NSP8-based peptide treatment. Peptide treatment against the (A) L-clade and (B) XBB Omicron subvariant, respectively. All results indicate triplicate repeats. The limit of detection (0.8 TCID_50_/mL) is indicated by a dashed line for each representation. The *p* values were calculated using a two-way ANOVA with Dunnett’s multiple comparison test. ∗*p* < 0.5; ∗∗*p* < 0.01; ∗∗∗*p* < 0.001; ∗∗∗∗*p* < 0.0001; ns, not significant.

The antiviral efficacy of the NSP8-based peptides was further evaluated using *in vitro* virus growth inhibition assays with recent SARS-CoV-2 strains. At first, Vero E6 cells were infected with a wild-type ancestral SARS-CoV-2 L-clade strain, and 1-h post-infection, peptides were added at varying concentrations during the media change. Viral replication was monitored by collecting cell culture supernatants at 12, 24, 48, and 72 h post-infection (hpi), followed by quantifying infectious virus titers using the 50% tissue culture ID_50_ (TCID_50_) assay. The results indicated a dose-dependent reduction in viral replication by all peptides, with N8-Pepα and N8-Pepα_cyc showing the strongest antiviral effects (Figure 4A). At higher concentrations, these peptides were associated with lower viral titers compared to the scrambled control peptide (N8-Pepα_sc) and mock-treated cells (Figures 4A and S6).

Given the global prevalence of the NSP12-P323L mutation, first reported in 2020^35^ and observed in over 99% of evaluated sequences from January 2023 to November 2024 (Figure S7), additional experiments were conducted to assess the impact of this mutation on peptide efficacy. Parallel assays were performed with the XBB1.17.1 Omicron subvariant, which carries the NSP12-P323L mutation known to enhance NSP12-NSP8 interactions. Despite this mutation, the NSP8-based peptides maintained similar dose-dependent inhibitory effects on viral replication, consistent with observations for the L-clade strain (Figure 4B). This consistency underscores the broad-spectrum antiviral activity of the peptides against diverse SARS-CoV-2 variants.

### NSP8-based peptides limit SARS-CoV-2 replication in mice and mitigate pulmonary damage

To evaluate the therapeutic and prophylactic efficacy of NSP8-based peptides against SARS-CoV-2 *in vivo*, a comprehensive study was conducted using Balb/c mice infected with two times the 50% mouse lethal dose (MLD_50_) of mouse-adapted SARS-CoV-2 (maSARS-CoV-2) (Figure S8). Mice were administered N8-Pepβ, N8-PepβD, N8-Pepα, N8-Pepα_cyc, or the scrambled control peptide N8-Pepα_sc intranasally at 25 mg/kg body weight (BW) in two sequential doses. To test treatment effectiveness under varying conditions, three subgroups (*n* = 14/subgroup) were established based on the timing of peptide administration relative to infection: a prophylactic group (−1 days post-infection [dpi]), a concurrent treatment group (0 dpi), and a post-exposure group (+1 dpi). Infection progression was monitored daily by assessing clinical signs, BW, coat condition, and activity levels. At 3, 5, and 7 dpi, viral titers in lung tissues and nasal turbinates were measured from sacrificed mice.

**Figure 5:**
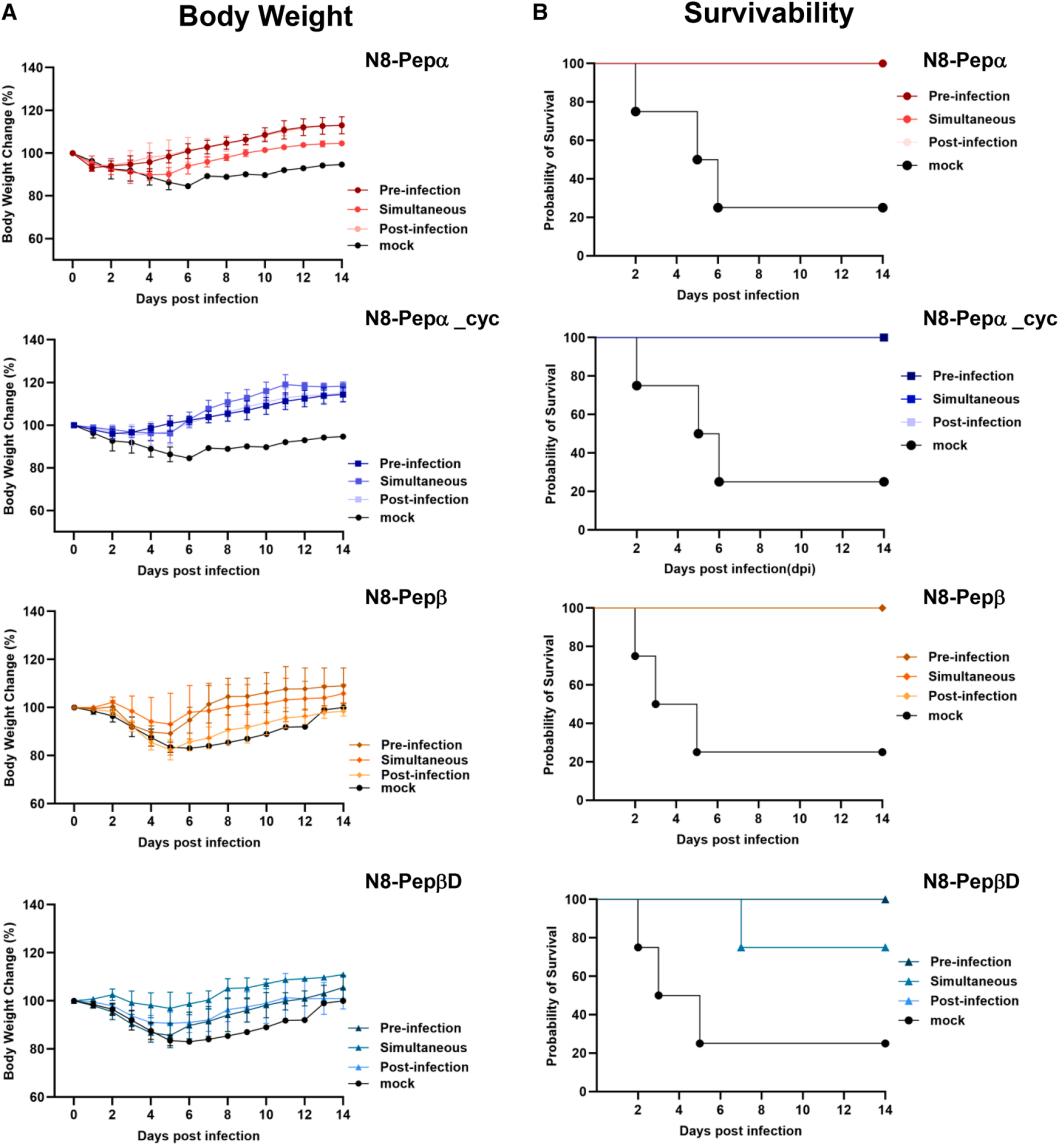
Enhanced recovery and survival in SARS-CoV-2-infected mice treated with NSP8 peptides Balb/c mice (*n* = 14/group) were inoculated intranasally with 2 LD_50_ of maSARS-CoV-2 (L = clade). Clinical manifestations of infection, body weight changes, and survivability were monitored for 14 days post-infection (dpi). (A and B) NSP8-based peptide treatment groups’ survivability and body weight changes.

BW analysis revealed that mock-treated and N8-Pepα_sc-treated mice experienced significant weight loss (10%–20%) by 3–6 dpi, while minimal or no weight loss occurred in mice treated with N8-Pepβ, N8-PepβD, N8-Pepα, or N8-Pepα_cyc (Figure 5A). Survival rates were notably higher in peptide-treated groups compared to controls (Figures 5B and S9). Notably, all mice treated with N8-Pepα, N8-Pepα_cyc, or N8-Pepβ exhibited 100% survival, with only mild to moderate clinical signs of disease. In the N8-PepβD group, survival was 100% in prophylactic and concurrent treatment subgroups, while a 20% mortality rate was observed in the post-exposure group. By contrast, mortality reached 75% in mock-treated and N8-Pepα_sc-treated mice by 5 and 6 dpi, respectively, accompanied by severe disease symptoms (Figure S9). These findings indicate significant protection against SARS-CoV-2 replication and disease severity in peptide-treated mice.

**Figure 6:**
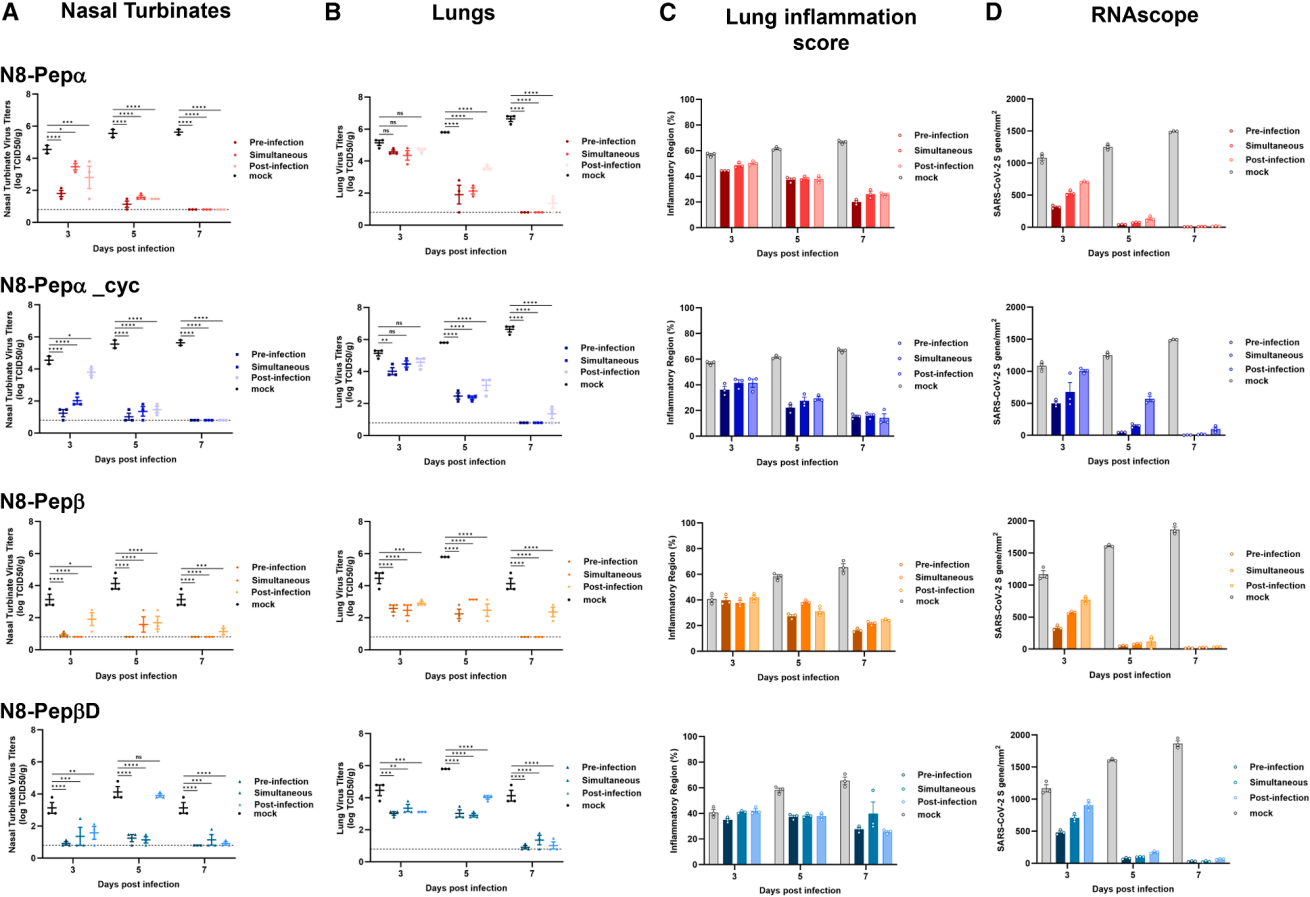
NSP8-based peptides limit SARS-CoV-2 replication in mouse respiratory tracts Groups of mice (*n* = 3/group) were sacrificed at 3, 5, and 7 dpi. The infectious virus titers in (A) nasal turbinates, (B) lung tissues among mice treated with either N8-Pepα, N8-Pepα_cyc, N8-Pepβ, or N8-PepβD administered prophylactically, simultaneous with infection, or post-infection, determining the TCID_50_ titers in Vero E6 cells. A dashed line indicates the limit of detection (0.8 TCID_50_/g). Inflammation scoring was performed using the H&E-stained lung sections from different NSP8-based treatment groups (C) and RNAscope analysis (D), both use the QuPath image analysis software. The *p* values were calculated using a two-way ANOVA with Dunnett’s multiple comparison test. ∗*p* < 0.5; ∗∗*p* < 0.01; ∗∗∗*p* < 0.001; ∗∗∗∗*p* < 0.0001.

Viral titration demonstrated that treatment with N8-Pepα, N8-Pepα_cyc, N8-Pepβ, and N8-PepβD significantly reduced viral loads in both the nasal turbinates and lungs compared to controls (Figure 6). Prophylactic administration (−1 dpi) was particularly effective, leading to earlier and more robust suppression of viral replication, with accelerated viral clearance observed in nasal turbinates (Figure 6A). Similar reductions were noted in lung tissue, where viral titers in peptide-treated groups were markedly lower than in mock-treated or N8-Pepα_sc-treated groups (Figure 6B). To further evaluate the antiviral activity of the peptides, we tested their effects against the SARS-CoV-2 XBB.1.17.1 variant (Figure S10). While infection with this strain did not cause mortality in the untreated control group, peptide treatment resulted in significantly faster and earlier clearance of infectious viruses from both nasal turbinates and lungs. These findings underscore the efficacy of the peptides in suppressing viral replication and promoting infection resolution, even in cases where severe disease or mortality is not induced by the XBB.1.17.1 variant.

Histopathological examination of lung tissues revealed that peptide treatment mitigated SARS-CoV-2-associated pulmonary damage. Lungs from mock-treated mice exhibited extensive inflammation and structural damage, while peptide-treated mice displayed only mild to moderate inflammation (Figures 6C and S11). RNAscope *in situ* hybridization confirmed a substantial reduction in SARS-CoV-2-infected cells in peptide-treated mice (Figures 6D and S12). In contrast, lungs from N8-Pepα_sc-treated mice showed more severe pathology, including increased inflammatory infiltration and higher numbers of infected cells (Figures S11 and S12). These results demonstrate the therapeutic and prophylactic potential of NSP8-based peptides *in vivo*, effectively reducing viral replication, mitigating disease progression, and limiting pulmonary damage.

### PK properties of NSP8-based peptides

**Figure 7:**
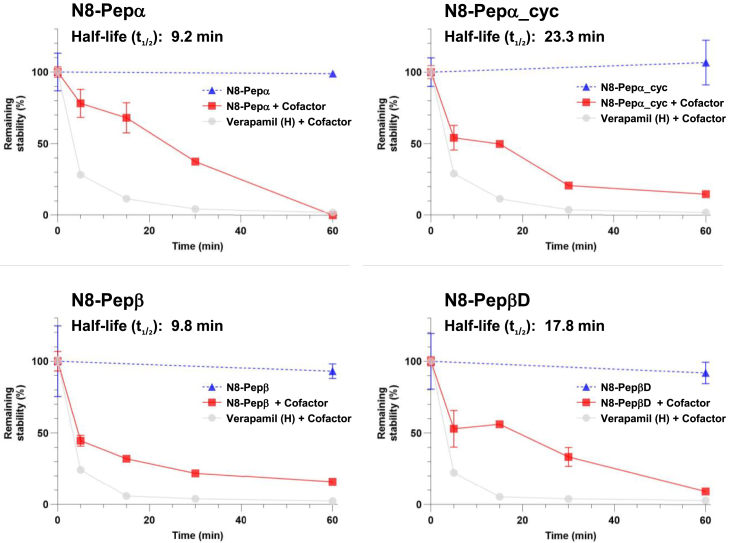
Liver microsomal stability of the NSP8-based peptides The stability of N8-Pepα, N8-Pepα_cyc, N8-Pepβ, and N8-PepβD was assessed in mouse liver microsomes over five time points (0, 5, 15, 30, and 60 min) to determine their half-life and clearance rates. The microsomal clearance rate was then converted to hepatic clearance to evaluate overall metabolic stability. Peptides were tested at a final concentration of 10 μM, and their stability was quantified over time.

To evaluate the therapeutic potential of NSP8-based peptides, we assessed their plasma and liver microsomal stability in mouse models to gain insights into their pharmacokinetic (PK) properties. Plasma stability assays (Table S3) revealed that all four peptides (N8-Pepβ, N8-PepβD, N8-Pepα, and N8-Pepα_cyc) underwent rapid degradation, with a short half-life of approximately 3 min and complete loss of detectable peptide within 20 min. In contrast, liver microsomal stability assays (Figure 7; Table S3) revealed that the N8-Pepα_cyc had the longest half-life (23.3 min), followed by N8-PepβD (17.8 min), while N8-Pepα and N8-Pepβ had half-lives of between 9.2 and 9.8 min. These results suggest that structural features, including cyclization, enhance metabolic resistance within cells.

### Mechanisms of NSP8-based peptide inhibition of SARS-CoV-2 replication

**Figure 8:**
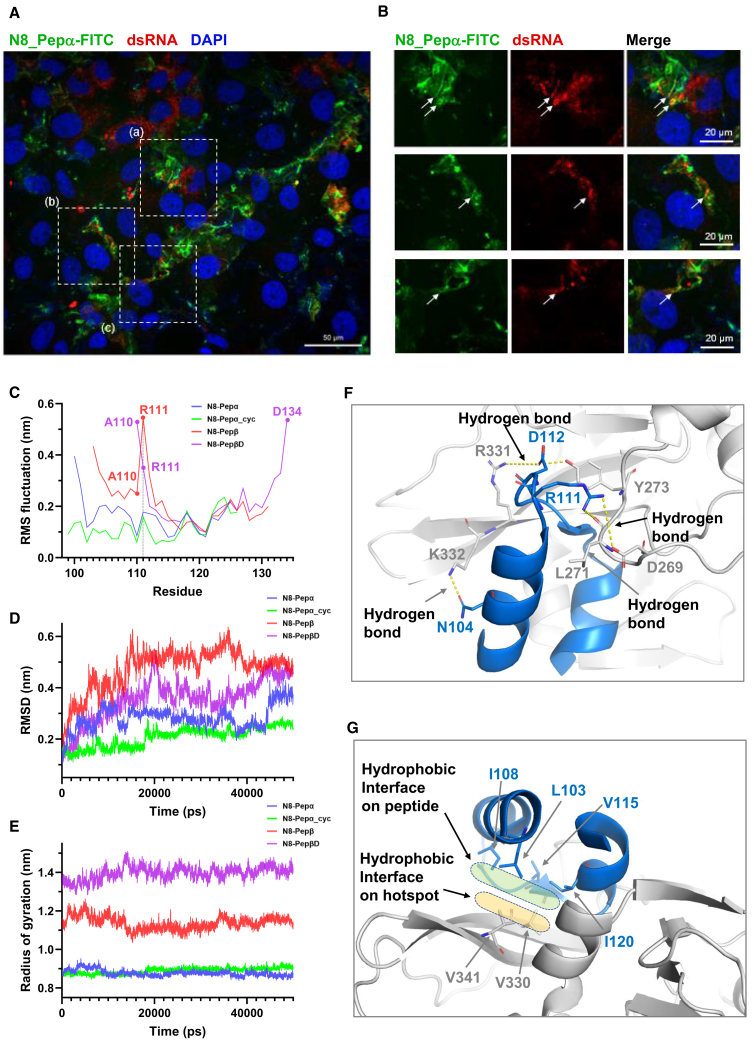
Molecular mechanism for inhibition by NSP8-based peptides (A) Immunofluorescence images showing the colocalization of N8_NE-FITC (green) with double-stranded RNA (dsRNA, red) in cells. The nucleus is stained with DAPI (blue). Regions of interest (a, b, c) indicate areas with prominent colocalization. Scale bar: 50 μm. (B) Magnified view of the colocalization between N8_Pepα-FITC and dsRNA. Arrows indicate the sites of interaction. Scale bar: 20 μm. (C) RMSF results for peptides in molecular dynamics simulations of the NSP8-based peptide-NSP12 complex. The *x* axis represents the residue numbers of the peptides, and the *y* axis shows fluctuation on a nanometer scale. To maintain consistency across all graphs (C–E), the peptides are represented with the same color scheme: N8-Pepα in blue, N8-Pepα in green, N8-Pepβ in red, and N8-PepβD in purple. (D) RMSD results for peptides in molecular dynamics simulations of the NSP8-based peptide-NSP12 complex. The *x* axis represents the simulation time in picoseconds, and the *y* axis shows deviation on a nanometer scale. (E) Radius of gyration (*Rg*) results for peptides in molecular dynamics simulations of the NSP8-based peptide-NSP12 complex. The *x* axis represents the simulation time in picoseconds, and the *y* axis shows the *Rg* on a nanometer scale. (F) Binding site of two α helices. NSP12 and the peptide are depicted in gray and blue, respectively, with residues involved in hydrogen bonding shown as sticks. (G) Hydrophobic interface between NSP12 and the peptide formed by the two α helices. Hydrophobic regions are highlighted with light green and ivory ovals.

Our studies demonstrated that the NSP8-based peptides—N8-Pepβ, N8-PepβD, N8-Pepα, and N8-Pepα_cyc—effectively inhibit viral replication. To elucidate their mechanisms of action, we investigated peptide interactions with the viral replication machinery. Using immunofluorescence assays with anti-double-stranded RNA (dsRNA) and anti-TAT antibodies, we confirmed the cellular localization and activity of these peptides. The TAT fusion ensured efficient cellular entry, as indicated during replication, colocalization of the peptides with the NSP12-NSP8-NSP7 complex was observed (Figure 8A). These dsRNA intermediates, along with the peptides, were found within structures consistent with double-membrane vesicles (Figure 8B), which are known to house SARS-CoV-2 replication organelles derived from the host endoplasmic reticulum.^36^ These observations suggest that NSP8-based peptides inhibit viral replication by binding directly to the RdRp complex and interfering with its function.

To gain further insight into the structural features of NSP8-based peptides, we performed molecular dynamics (MD) simulations of peptide-NSP12 complexes, including NSP12-N8-Pepα, NSP12-N8-Pepα_cyc, NSP12-N8-Pepβ, and NSP12-N8-PepβD. Root-mean-square fluctuation (RMSF) analysis showed that N8-Pepα and N8-Pepα_cyc exhibited lower flexibility at residues such as R111 and D112, which are potentially involved in NSP12 binding (Figure 8C). In contrast, N8-Pepβ and N8-PepβD exhibited higher fluctuations, particularly at the termini. These differences may reflect reduced conformational stability in β strand-containing peptides, potentially related to the absence of a second α helix. Root-mean-square deviation (RMSD) values were lower for the α helical peptides (Figure 8D), and radius of gyration (*Rg*) values suggested a more compact structure compared to N8-Pepβ and N8-PepβD (Figure 8E). Collectively, these findings support the notion that dual α helical conformations may contribute to greater structural stability and NSP12 binding.

## Discussion

The ongoing global threat posed by SARS-CoV-2 underscores the need for innovative therapeutic approaches targeting conserved viral mechanisms. The RdRp complex, composed of NSP12, NSP8, and NSP7, is crucial for SARS-CoV-2 replication, making it an ideal target for intervention. Our study explored the potential of NSP8-based peptides designed to disrupt the essential interaction between NSP12 and NSP8, which is critical for RdRp assembly and catalytic activity. NSP8 not only serves as a cofactor for NSP12 but it also interacts with other viral components that facilitate efficient replication. For instance, the total deletion of NSP8 in murine hepatitis virus resulted in impaired viral synthesis and a lethal phenotype.^37^ In addition, Campagnola et al. showed that double deletion of the N-terminal helix region of the NSP8, nsp8-Δ75 with nsp8L7-Δ59, causes insufficient formation of active complex.^38^

Through MD and structural analyses, we identified a key interaction hotspot at the NSP12-NSP8 interface, involving residues 111–125 of NSP8. These residues form strong hydrophobic and hydrogen bond interactions with the NSP12 hotspot (Figures 8F and 8G), indicating that peptides derived from this region could effectively reduce RdRp activity by disrupting the NSP12-NSP8 interaction. This aligns with prior studies, where mutations in NSP8 (e.g., K58A, D99A) and NSP12 (e.g., V330S, V341S) impaired the NSP12-NSP8 interaction and reduced polymerase activity.^26^^,^^29^ N8-Pepα and N8-Pepα_cyc demonstrated the strongest inhibition of RdRp activity in the minigenome assay and showed the highest direct binding affinity in SPR analyses. These data, together with the MD simulations, suggest that maintaining a stable dual α helical conformation contributes to effective NSP12 binding and functional inhibition.

SPR analysis substantiated the direct binding of N8-Pepα, N8-Pepα_cyc, and N8-Pepβ to NSP12, in contrast to N8-PepβD, which exhibited low *R*_*max*_ and inconsistent kinetic parameters, indicating weak or transient interaction (Figure S2). Despite weak binding or transient binding in SPR, N8-PepβD showed partial inhibition in MST, possibly due to indirect interference with the NSP12-NSP8 complex formation. These results highlight the importance of evaluating both direct binding and functional inhibition independently and underscore the superior structural and biophysical characteristics of dual α helical peptides, particularly N8-Pepα and its cyclized derivative.

Further validation *in vivo* using a Balb/c mouse model showed the therapeutic and prophylactic potential of these peptides. Mice treated with N8-Pepα and N8-Pepα_cyc achieved 100% survival after lethal SARS-CoV-2 challenge, along with significant reductions in viral titers in nasal turbinates and lungs. Early suppression of viral replication is critical for limiting viral spread and mitigating disease progression, underscoring the translational promise of these peptides. The observed discrepancy in peptide efficacy between the minigenome assay and *in vitro* virus replication studies can be attributed to differences in peptide stability and delivery context. In the minigenome assay, which mimics plasma-like conditions without continuous exposure, peptides are subject to rapid degradation, necessitating higher concentrations to achieve RdRp inhibition. In contrast, repeated intranasal administration *in vitro* and *in vivo* likely facilitates sustained local peptide accumulation at primary replication sites, such as the nasal mucosa and lungs. This local retention may enable lower concentrations to exert antiviral effects over time. The enhanced metabolic stability of N8-Pepα_cyc in liver microsomes further supports its therapeutic durability in target tissues. These findings underscore the importance of peptide delivery routes and metabolic stability in determining antiviral efficacy.

Our data also indicated that peptides incorporating β strand regions showed weaker inhibitory effects, likely due to the inherent limitations of β strands in maintaining stable interactions. In contrast, α helices, stabilized by intramolecular hydrogen bonds, are more structurally favorable for peptide binding. The dual α helix structure in NSP8-derived peptides appears to enhance binding stability via additional interactions such as hydrogen bonds or salt bridges between residues like NSP8-D112, NSP8-R111, or NSP12-K332. MD simulations, particularly *Rg* analyses, supported these findings, showing that β strand-containing peptides exhibited weaker binding and less structural compactness. While these simulation data are consistent with our structural interpretation, further biochemical validation is necessary to confirm the functional roles of these interactions.

Our findings suggest that N8-Pepα, N8-Pepα_cyc, and their derivatives are promising antiviral agents targeting SARS-CoV-2 replication by disrupting the NSP12-NSP8 interaction, a conserved component of the viral RdRp complex. The development of SARS-CoV-2 therapeutics has advanced significantly, with a variety of approved and investigational agents targeting distinct stages of the viral life cycle. Small-molecule inhibitors, such as remdesivir and molnupiravir, offer advantages like oral bioavailability and ease of manufacturing. However, these agents may face challenges such as off-target effects and the emergence of resistant variants. In contrast, peptide-based antivirals exhibit several favorable properties, including high target specificity, the ability to disrupt protein-protein interactions, and biocompatibility, making them attractive candidates for targeting conserved viral interfaces. For instance, peptide therapeutics like EK1C4 (fusion inhibitor) and GC376 (Mpro inhibitor) have shown potent antiviral effects in preclinical models. Moreover, existing peptide-based drugs for other viral infections, such as enfuvirtide (HIV) and boceprevir or telaprevir (hepatitis C virus),^39^^,^^40^^,^^41^ underscore the clinical utility of this drug class. Nonetheless, peptide drugs are often limited by poor stability *in vivo* and low cell permeability. Compared to monoclonal antibodies (e.g., sotrovimab),^42^ which are expensive, require parenteral administration, and may lose efficacy against new variants, peptide drugs offer a more cost-effective and modular therapeutic platform. These features support their inclusion in diversified antiviral strategies, particularly for targeting intracellular processes such as RNA replication. Notably, no therapeutic strategies to date have targeted the NSP12-NSP8-NSP7 complex, a critical component for SARS-CoV-2 replication. Our study is the first to explore this novel approach using NSP8-derived peptides, filling a critical gap in existing therapeutics. Given the high conservation of RdRp complexes across alphacoronaviruses (e.g., HCoV-229E, NL63) and betacoronaviruses (e.g., SARS-CoV, Middle East respiratory syndrome-CoV), our approach holds promise for developing broad-spectrum antiviral agents against a range of coronavirus-related diseases, including those with pandemic potential.

Despite the promise of NSP8-based peptides, certain challenges remain. Cyclization improved peptide stability but did not consistently enhance efficacy, as seen with N8-Pepα_cyc. Its slight weaker binding affinity to N8-Pepα and N8-Pepβ suggested that cyclization might limit the flexibility needed for optimal α helix formation. Future strategies could include lactam-based cross-links or macrocyclization to stabilize α helices while maintaining functional dynamics.^43^ Additionally, enhancing peptide delivery and bioavailability is essential for clinical application. Alternative CPPs or optimized CPP placement, such as modified TAT promoters or poly-Arg sequences, could improve intracellular delivery, particularly to lung tissues.^44^^,^^45^^,^^46^ These strategies, combined with structural optimizations, are crucial for advancing NSP8-based peptides from preclinical research to clinical use.

An additional potential limitation of this study is the possibility of escape mutants arising from incomplete viral inhibition. To address this risk, refinement strategies involving advanced modifications, such as hydrocarbon stapling, could enhance peptide stability, cell permeability, and binding affinity to the NSP12-NSP8 interface, potentially leading to more complete viral suppression. Furthermore, combination therapy with complementary antiviral agents, such as nucleoside analogs (e.g., remdesivir), may help mitigate resistance development by targeting distinct mechanisms within the viral replication cycle.

In conclusion, our study demonstrated that NSP8-based peptides effectively inhibit SARS-CoV-2 replication by targeting the NSP12-NSP8 interaction, with α-helical peptides showing superior efficacy. The antiviral activity of these peptides against emerging variants underscores their relevance in the context of viral evolution. As SARS-CoV-2 continues to evolve, targeting conserved viral machinery like the RdRp complex will be critical for future therapeutic strategies. Our work provides a foundation for further optimization and clinical translation of peptide-based antivirals for SARS-CoV-2 and related coronaviruses.

## Materials and methods

### Protein preparation

The Bacmid encoding SARS-CoV-2 NSP12 with N-terminal tagged 6× His were transfected to Sf9 cells and cultured based on the manufacturer’s instruction (Thermo Fisher). The recombinant baculovirus encoding NSP12 was infected with 200 mL Hifive cells (Thermo Fisher) and incubated at 27°C for 3 days. Cells were harvested by centrifugation and resuspended in 25 mL 25 mM HEPES pH 7.4, 300 mM sodium chloride, 1 mM magnesium chloride, and 5 mM β-mercaptoethanol by sonication. After centrifugation at 25,000 × *g* for 40 min and passing through a 0.45-μm filter, cell lysate was loaded onto HisPur Cobalt Resin (Thermo Fisher) and eluted with an elution buffer containing 300 mM imidazole. Eluted protein was concentrated and further purified by size exclusion chromatography using a Superdex200 column in the same buffer for resuspension.

Recombinant NSP7 and NSP8 proteins were expressed and purified using pET-11 vector (Novagen). Transformed BL21(DE3) cells were induced by 1.0 mM isopropyl β-d-1-thiogalactopyranoside and cultured in an LB medium for 18 h at 18°C. The cells were harvested, lysed with PBS buffer by sonication, and cleared by centrifugation. Finally, the recombinant NSP7 and NSP8 were purified using HisPur Cobalt Resin (Thermo Fisher) and size exclusion chromatography using a Superdex200 column with PBS buffer, sequentially.

### Peptide docking to NSP12

Peptide docking simulations were performed using the HADDOCK web server (HADDOCK 2.4).^32^^,^^33^ NSP8-based peptides were docked to NSP12 structure extracted from NSP12-NSP8-NSP7-RNA complex structure (PDB: 6YYT). Docking simulations were conducted using active residues based on the interface between NSP12 and NSP8. The HADDOCK analysis provided binding scores for the NSP12-peptide complexes. The HADDOCK score is calculated as a weighted sum of several energy terms, including van der Waals energy, electrostatic energy, desolvation energy, and distance restraints energy (http://bonvinlab.org/software/haddock2.4/scoring/). PyMOL Molecular Graphics System 2.5.5 version was used to visualize the complex structure and docking results.

### MST assay

Purified recombinant proteins were concentrated to the maximum feasible levels. Then, proteins were used to desired concentrations as PBS with Tween 20 buffer. The recombinant NSP12 protein was labeled with cytidine-5 dye using the Monolith His-Tag Labeling Kit (NanoTemper Technologies) and diluted to 20 nM. Stock ligands were prepared by combining NSP8 (16 μM), NSP7 (8 μM), and RNA (8 μM). Binding affinity changes were measured with or without the peptide (3:1 ratio, 24 μM) added to the stock ligand according to the manufacturer’s instructions (NanoTemper Technologies). The thermophoretic traces were monitored and the *K*_D_ was calculated by analyzing the change in fluorescence (*ΔF*_*norm*_). *F*_*norm*_ was obtained by dividing the fluorescence measured in the heated state (*F*_*1*_) by that in the cold state (*F*_*0*_). *K*_D_ values were determined based on fluorescence intensity changes (*ΔF*_*norm*_) due to the thermophoretic effect during the interaction of fluorescence-labeled NSP12 with the ligand mixture (NSP7 + NSP8 + RNA). Binding affinities were analyzed and plotted using MO.Affinity Analysis software (NanoTemper Technologies).

### SPR analysis

SPR experiments were performed using a Biacore T200 system (Cytiva, formerly GE Healthcare) at the Research Solution Center of the Institute for Basic Science (IBS). Four peptides were immobilized onto a CM5 sensor chip via the lysine residue within the TAT CPP sequence, achieving approximately 140 response units for each peptide.

The running buffer was HBS-EP (10 mM HEPES, 150 mM NaCl, 3 mM EDTA, and 0.05% surfactant P20, pH 7.4). The interaction was measured by injecting the analyte NSP12 protein at concentrations of 1, 3, 10, 30, 100, and 300 nM at a flow rate of 50 μL/min. Each association phase lasted 180 s, followed by a dissociation phase of 1,200 s and a final stabilization period of 180 s.

Sensorgrams were fitted using a 1:1 Langmuir binding model in the Biacore Evaluation software. From these fits, kinetic parameter values, including the association rate constant (*k*_*on*_), dissociation rate constant (*k*_*off*_), and equilibrium dissociation constant (*K*_*D*_) were calculated.

### Cell-based SARS-CoV-2 RdRp activity reporter assay

The cell-based reporter system utilized a bicistronic construct containing firefly luciferase and Nano-Glo Luciferase flanked by SARS-CoV-2 UTRs and the hepatitis D virus ribozyme sequence, following the methodology described in our previous research.^5^ The SARS-CoV-2 reporter and NSP12, NSP7, and NSP8 genes were cloned into pcDNA3.1(+) plasmid vectors. We transfected 293T cells with plasmids using TransIT-LT1 reagent and incubated at 37°C. Luciferase activities were measured using the Nano-Glo Dual-Luciferase Reporter Assay System (Promega) as per the manufacturer’s protocol. The RdRp activity reporter assay was conducted in triplicate and independently repeated three times.

### Cytotoxicity assay

The cytotoxicity of test samples was assessed in Vero-E6 cells using the MTT assay, following the manufacturer’s protocol for the CellTiter 96 Non-radioactive Cell Proliferation Assay (Promega). Briefly, HEK-293T cells were seeded in 96-well plates at a density of 2 × 10^4^ cells per well and incubated for 24 h. Following this, cells were treated with test samples at concentrations ranging from 0.2 to 40 μM and incubated for an additional 24 h. After incubation, 15 μL of MTT reagent (dye solution) was added to each well, and the cells were further incubated for 4 h. Subsequently, 100 μL of solubilization/stop solution was added to dissolve the formazan crystals. The optical density was measured at 570 nm, and statistical significance was determined by comparing the results to those of the mock-treated cells.

### Cells and *in vitro* virus growth kinetics

Vero E6 and HEK-293T cells were cultured in DMEM supplemented with 10% fetal bovine serum and 1% penicillin-streptomycin. To determine viral growth inhibitory effects of the peptides, Vero E6 cells were inoculated with each virus at 0.001 multiplicity of infection, and after incubation for 1 h, the cells were changed to media containing varied concentrations of each peptide. Virus culture media was harvested at 12, 24, 48, and 72 hpi and were used to titrate Vero cells to determine the TCID_50_. The viral growth kinetics was performed in triplicate, and each experiment was independently repeated at least three times.

### Virus titration assay

For the titration of viruses, mice tissue homogenate and cell supernatants were serially diluted with DMEM and added to Vero E6 in a 96-well plate. After 1 h of infection, cells were washed with PBS and incubated for 3 days at 37°C under 5% CO_2_. Then, cytopathic effects were observed to calculate the TCID_50_/mL.

### Animal experiments

The animal experiments were conducted ethically after approval from the Institutional Animal Care and Use Committee (IACUC) of the IBS (approval no. IBS-2024-027). All experiments adhered to relevant guidelines and regulations. Baseline BWs were measured prior to infection, and survival rates and subsequent BWs were recorded 14 dpi. For *in vivo* efficacy testing, 4-week-old female BALB/c mice were intranasally infected with 2 MLD_50_ of a mouse-adapted SARS-CoV-2 Wuhan strain (maS2p30), generated via 30 serial lung-to-lung passages. The resulting strain carries the mutations N501Y, K417N, and K498H, conferring enhanced pathogenicity and lethality, as previously described.^47^^,^^48^^,^^49^ All procedures were conducted in a certified ABSL-3 facility under an IACUC-approved protocol (IBS-2024-029). The peptide-treated groups (N8-PEPΑ, N8_NEcyc, or N8_NEsc) received a total dose of 25 mg/kg BW administered intranasally in two sequential doses. Mice were subdivided into three subgroups (*n* = 14/subgroup) based on the timing of the initial peptide administration: 1 day prior to infection (−1 dpi), at the time of infection (0 dpi), and 1 dpi. A mock treatment group (0.5% DMSO in PBS) was included as a control. Three mice per subgroup were euthanized at 3, 5, and 7 dpi, and nasal turbinates and lungs were harvested to assess viral growth kinetics using the TCID_50_ assay.

### Lung histopathology

To quantify the number of inflammatory cells infiltrating the lung, sections were prepared and stained with hematoxylin and eosin (H&E) (Celltisbio). Whole-lung sections were digitally scanned at 40× magnification with an Axio Scan Z1 (Zeiss). Automated cell detection and classification into parenchymal cells, airway and vasculature cells, and inflammatory cells were performed based on the QuPath image training.^50^ The training dataset comprised at least 30 H&E-stained lung images, encompassing both normal and pathologically altered specimens.

### RNA scope *in situ* hybridization

SARS-CoV-2 RNA (Spike) was detected using the nCoV2019-2 probe (Advanced Cell Diagnostics, 848956) and visualized using RNAscope 2.5 HD Reagent Kit RED (Advanced Cell Diagnostics, 322360). According to the manufacturer’s instructions, mouse lung tissues were fixed in 4% neutral buffered formalin, embedded, sectioned, and counterstained with Gill’s hematoxylin no. 1 (Polysciences, 24242-1000). Slides were viewed using an Olympus IX 71 microscope with digital photomicrography (DP) controller software to capture images. For statistical analysis of *in situ* samples, positive foci (most likely resembling single positive cells) were counted manually for each field, and the standard deviation was calculated using GraphPad Prism version 9.4.1.

### PK assays

The metabolic stability of peptides (N8-Pepα, N8-Pepα_cyc, N8-Pepβ, and N8-PepβD) was assessed in mouse liver microsomes by WuJungBio Inc. Mouse liver microsomes (CD-1, pooled) were incubated with each peptide (10 μM final concentration) at 37°C with NADPH cofactor for 0, 5, 15, 30, and 60 min. Reactions were quenched and peptide concentrations were quantified using liquid chromatography-tandem mass spectrometry (LC-MS/MS) (AB Sciex QTRAP 6500+, Analyst software version 1.7.2). Clearance and half-life were calculated by non-compartmental analysis, and values were converted to hepatic clearance (CLint, liver) using standard scaling factors.

Plasma stability of the peptides was evaluated using mouse plasma (ICR, male, K2-EDTA) purchased from Innovative Research. Each peptide (10 μM final concentration) was incubated at 37°C for 0, 20, 40, 60, and 120 min. Samples were processed and analyzed by LC-MS/MS, as described above. Procaine was used as a positive control. Half-life (t_1/2_) values were calculated based on the degradation curve, and metabolic stability was classified as high (t_1/2_ < 30 min), moderate (30–120 min), or low (>120 min).

All *in vitro* stability experiments were performed in duplicate (*n* = 2). Since the standard deviation was less than 20% in all cases, no additional replicates were conducted.

### MD simulation

In this study, MD simulation was performed to evaluate the motions, fluctuations, and stability of peptide in the protein-peptide complexes using Gromacs-2024.4.^51^^,^^52^^,^^53^^,^^54^ The initial stages of protein-peptide complexes for MD simulation were derived from the best-scoring model of HADDOCK. The input topologies files for the protein-peptide complex were prepared with AMBER99SB-ILDN protein, nucleic AMBER94 parameters.^55^

The simulation system was defined within a cubic unit cell, ensuring that each model was centered in the box and positioned at least 1 nm away from its edges. The system was solvated using the simple point charge water model and neutralized with suitable counterions, such as sodium or chloride ions. Energy minimization was performed using the steepest descent algorithm with a stopping criterion of maximum force <1,000.0 kJ/mol/nm, a step size of 0.01 nm, and up to 50,000 steps.

Equilibration was carried out in two stages: an NVT equilibration for 100 ps at 300 K, followed by an NPT equilibration for 1,000 ps at 1 atm. Subsequently, a 50,000-ps production MD simulation was conducted under constant temperature and pressure. The GROMACS tools were utilized to calculate RMSD, RMSF, and *Rg*.

### Statistical analysis

Statistical analyses were performed using GraphPad Prism 9.3.1. Statistical details of our experiments and analyses can be found in each figure legend.

## Data availability

All relevant data supporting the findings of this study are included in the paper and its supplemental information. Additional datasets generated and analyzed during the present study are available from the corresponding author upon reasonable request.

## Acknowledgments

This work was supported by the 10.13039/501100010446Institute for Basic Science (grant no. IBS-R801-D1) and the 10.13039/501100001321National Research Foundation (NRF) funded by the Korean government (MSIT) (grant no. RS-2024-00400771) to Y.K.C. The three-dimensional illustration of the SARS-CoV-2 virion in the graphical abstract was obtained from the Centers for Disease Control and Prevention Public Health Image Library under identification no. 23312. We thank Veronica Falconieri Hays; the image was originally published in *Scientific American*. The remaining elements of the graphical abstract were created using BioRender.com (https://BioRender.com/tp0z3x8).

## Author contributions

Conceptualization and study design: M.A.B.C., J.-W.A., H.K., and Y.K.C. Performed experiments: M.A.B.C., J.-W.A., H.K., I.C., S.-G.J., R.R., H.-Y.J., M.Y., and S.C.M. Data analysis and interpretation: M.A.B.C., J.-W.A., H.K., and M.-S.S. Writing – drafted and collectively finalized: M.A.B.C., J.-W.A., H.K., and Y.K.C. Supervision: Y.K.C.

## Declaration of interests

The authors declare no competing interests.

## Supplemental information

Document S1. Figures S1–S13 and Tables S1–S3

Document S2. Article plus supplemental information
