# Supplementary material for: Therapeutic applications of interface-mimicking peptides for targeting the SARS-CoV-2 NSP12-NSP8 RdRp complex
Source: Mol Ther. 2025 May 27;33(9):4471–87. doi: 10.1016/j.ymthe.2025.05.028 (PMC12432892; doi:10.1016/j.ymthe.2025.05.028)
Supplement: Supplementary file 1 [file mmc1.pdf]

YMTHE, Volume *33*

Supplemental Information

Therapeutic applications of interface-mimicking peptides for targeting the SARS-CoV-2 NSP12-NSP8 RdRp complex

Mark Anthony B. Casel, Jae-Woo Ahn, Hyunjoon Kim, Isaac Choi, Seung-Gyu Jang, Rare Rollon, Ho-Young Ji, Mina Yu, Seong Cheol Min, Min-Suk Song, and Young Ki Choi

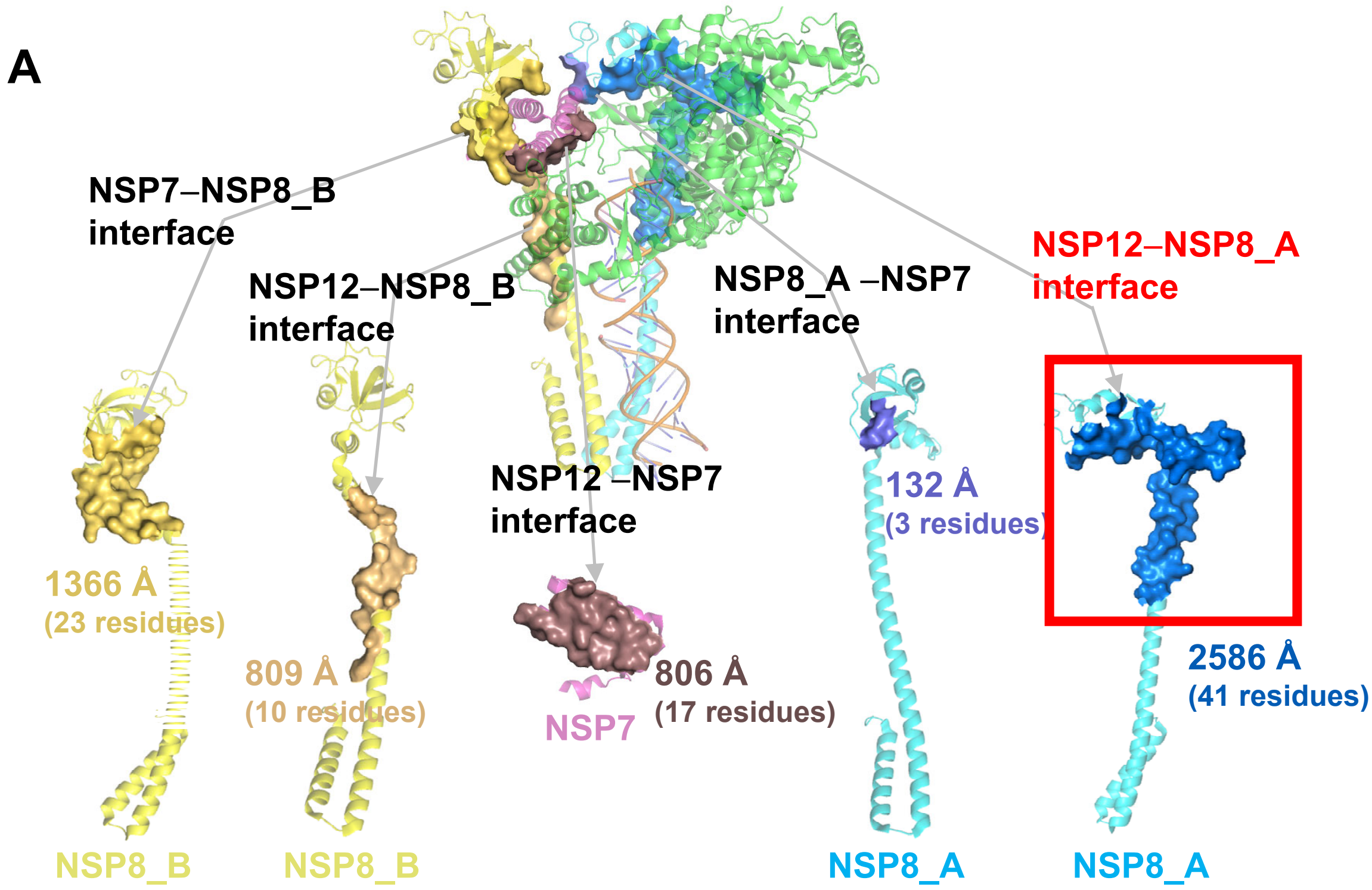

B

Chains	No. of interfaces residues	Interface area (Å²)	No. of salt bridges	No. of Hydrogen bonds	No. of non-bonded contacts
NSP12—NSP8_A	43 : 41	2435 : 2586	2	15	176
NSP12—NSP7	18 : 17	779 : 806	-	5	61
NSP12—NSP8_B	15 : 10	741 : 809	-	3	55
NSP8_A—NSP7	3 : 4	132 : 150	-	3	17
NSP7—NSP8_B	28 : 23	1362 : 1366	-	5	85

Figure S1. Interface display and statics of NSP12-NSP8-NSP7complex
(A) Comparison of the binding interface areas in the NSP12-NSP8-NSP7 complex (PDB code: 6YYT). The NSP12–NSP8_A interface, used as the target interface, is highlighted with a red box. (B) Interface statics of NSP12-NSP8-NSP7complex. The statistics of the target interface, NSP12-NSP8_A, are outlined in red.

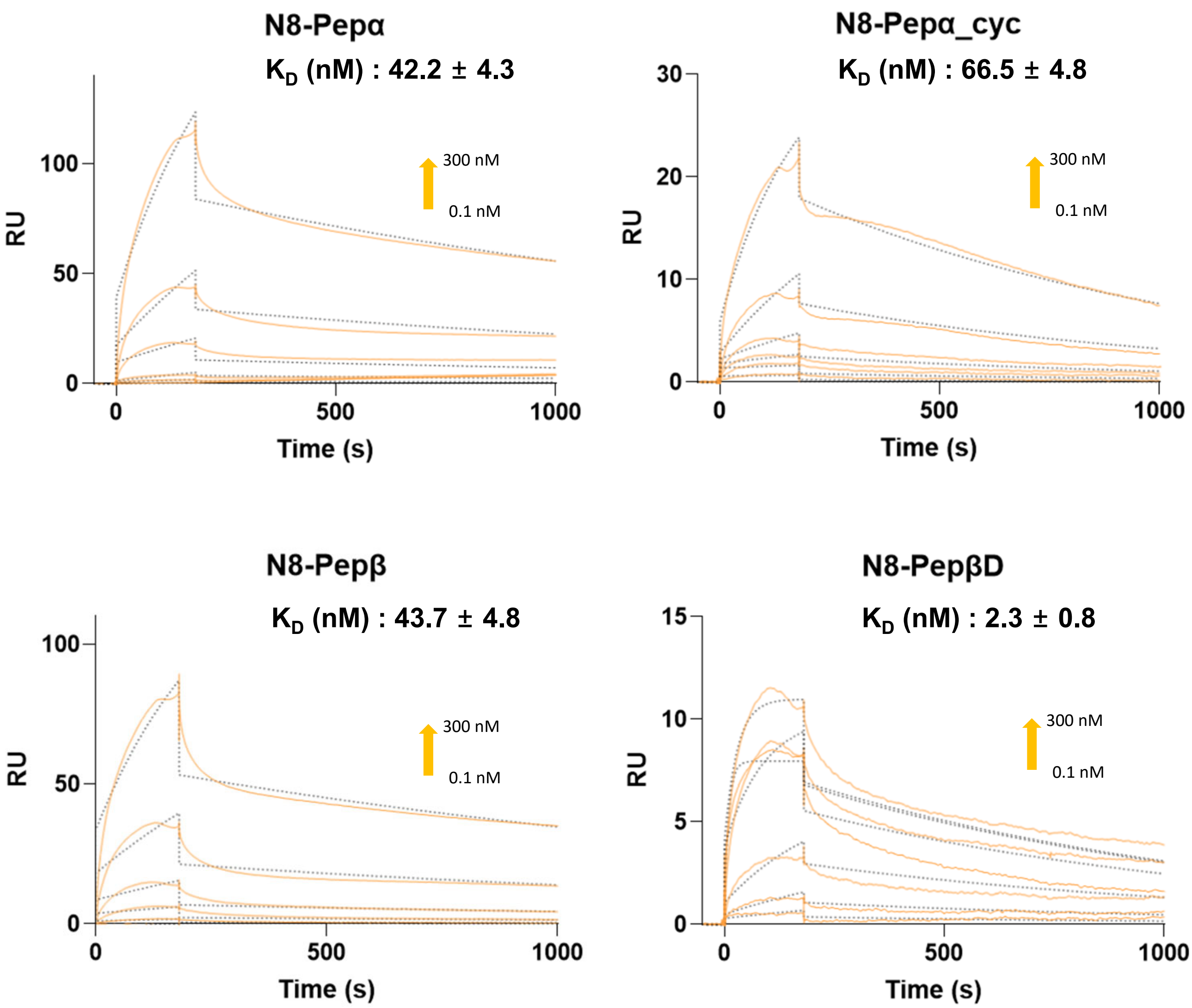

Figure S2. SPR sensorgrams showing the binding kinetics of NSP8-derived peptides to NSP12.
Representative SPR sensorgrams of N8-Pepα, N8-Pepα_cyc, N8-Pepβ, and N8-PepβD binding to immobilized NSP12. Increasing concentrations of peptides (0.1 to 300 nM) were injected to determine binding kinetics. The dissociation constant (K_D) for each peptide is indicated.

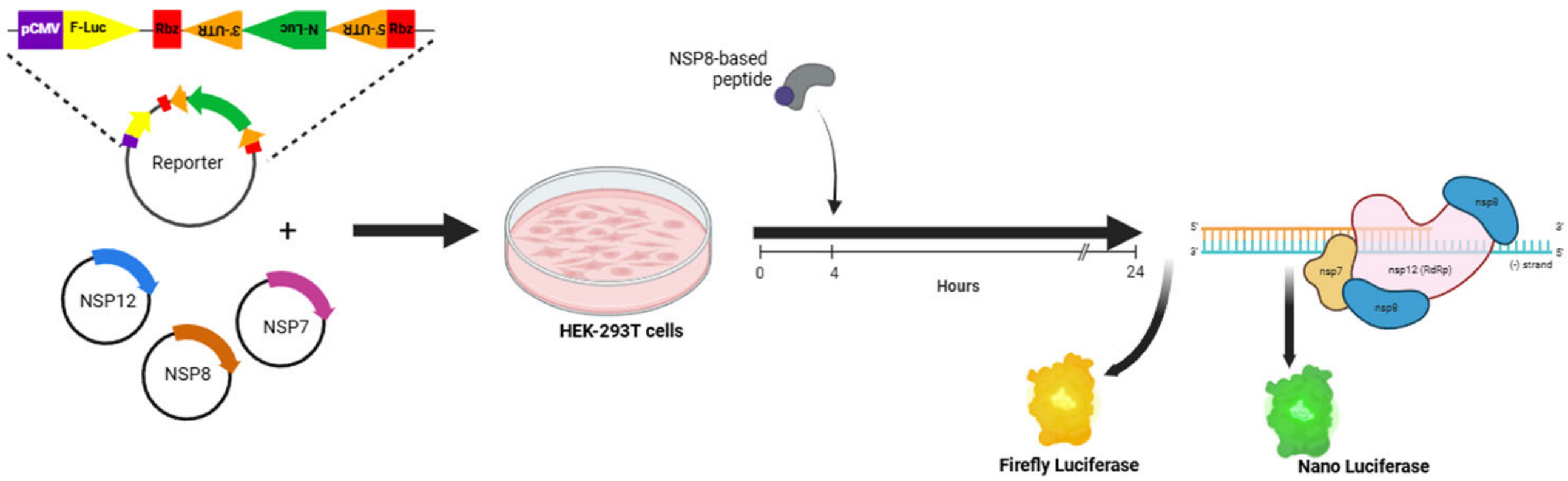

Figure S3. Schematic representation of antiviral screening using the SARS-CoV-2 minigenome assay.

HEK-29T cells were transiently transfected with the SARS-COV-2 RdRp reporter gene assay. At 4 hours post-transfection, cells were media changes containing varied concentrations of each NSP8-based peptide. NLuc activities were measured 24 hours post-transfection.

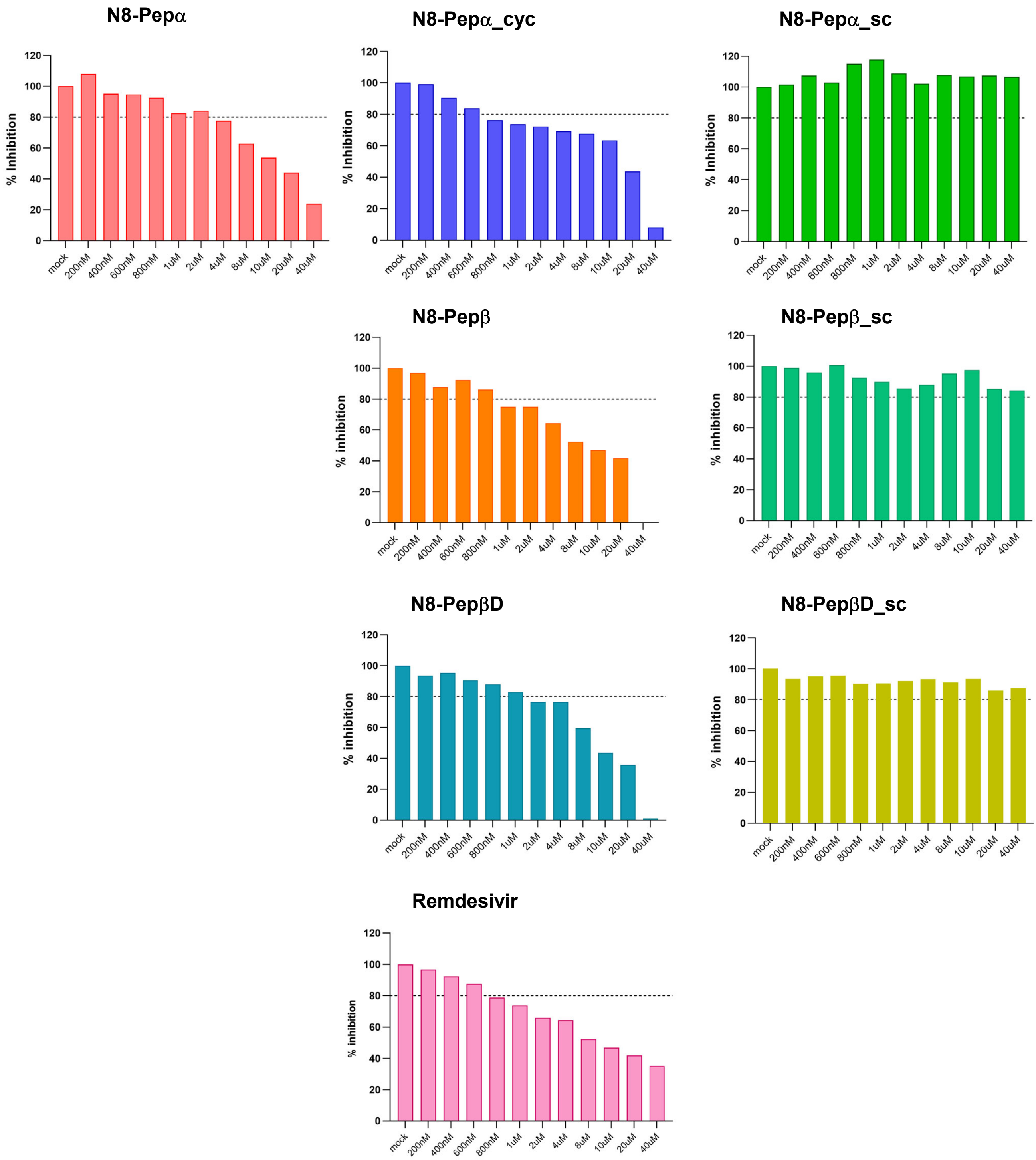

Figure S4. Minigenome assay of peptides

Minigenome assay results display normalized luciferase activity relative to untreated controls for various peptides: N8-Pepα, N8-Pepα_cyc, N8-Pepβ, N8-PepβD, the scrambled controls (N8-Pepα_sc, N8-Pepβ_sc, and N8-PepβD_sc), and Remdesivir (Positive Control).

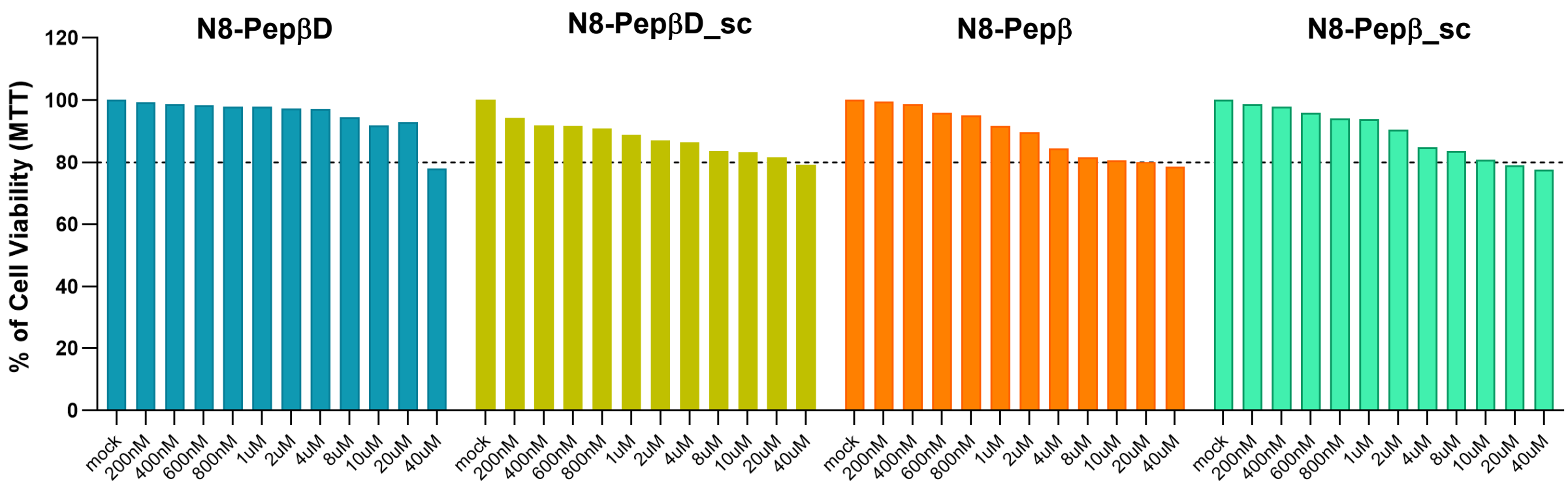

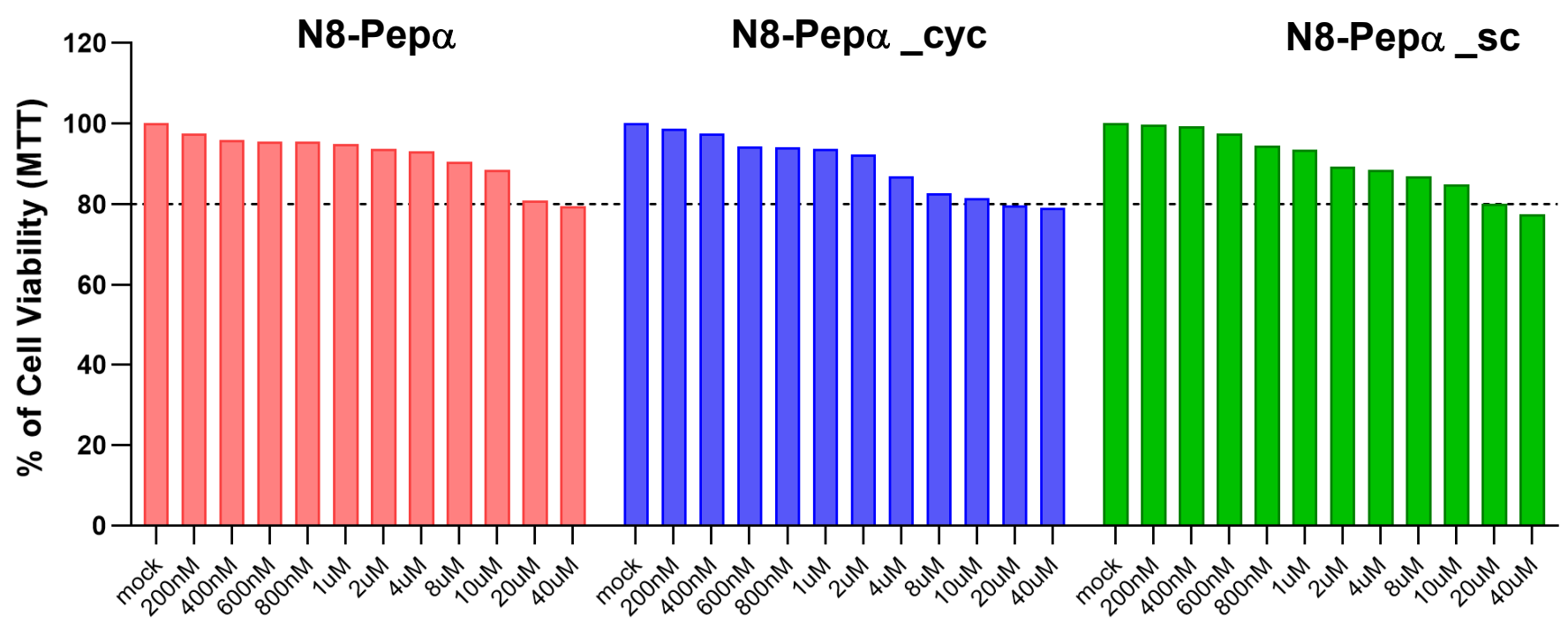

Figure S5. MTT assay of peptides including scrambled version
Vero-E6 cells were treated with NSP8-based peptides at concentrations ranging from 0.2 to 40 μM and incubated for an additional 24 hours. Cytotoxicity was evaluated using the MTT assay to assess the impact of the peptides on cell viability. According to IOS 10993-5 guidelines, cell viability above 80% indicates no cytotoxicity, 80%-60% suggests weak cytotoxicity, 60-40% moderate cytotoxicity, and below 40% signifies strong cytotoxicity.

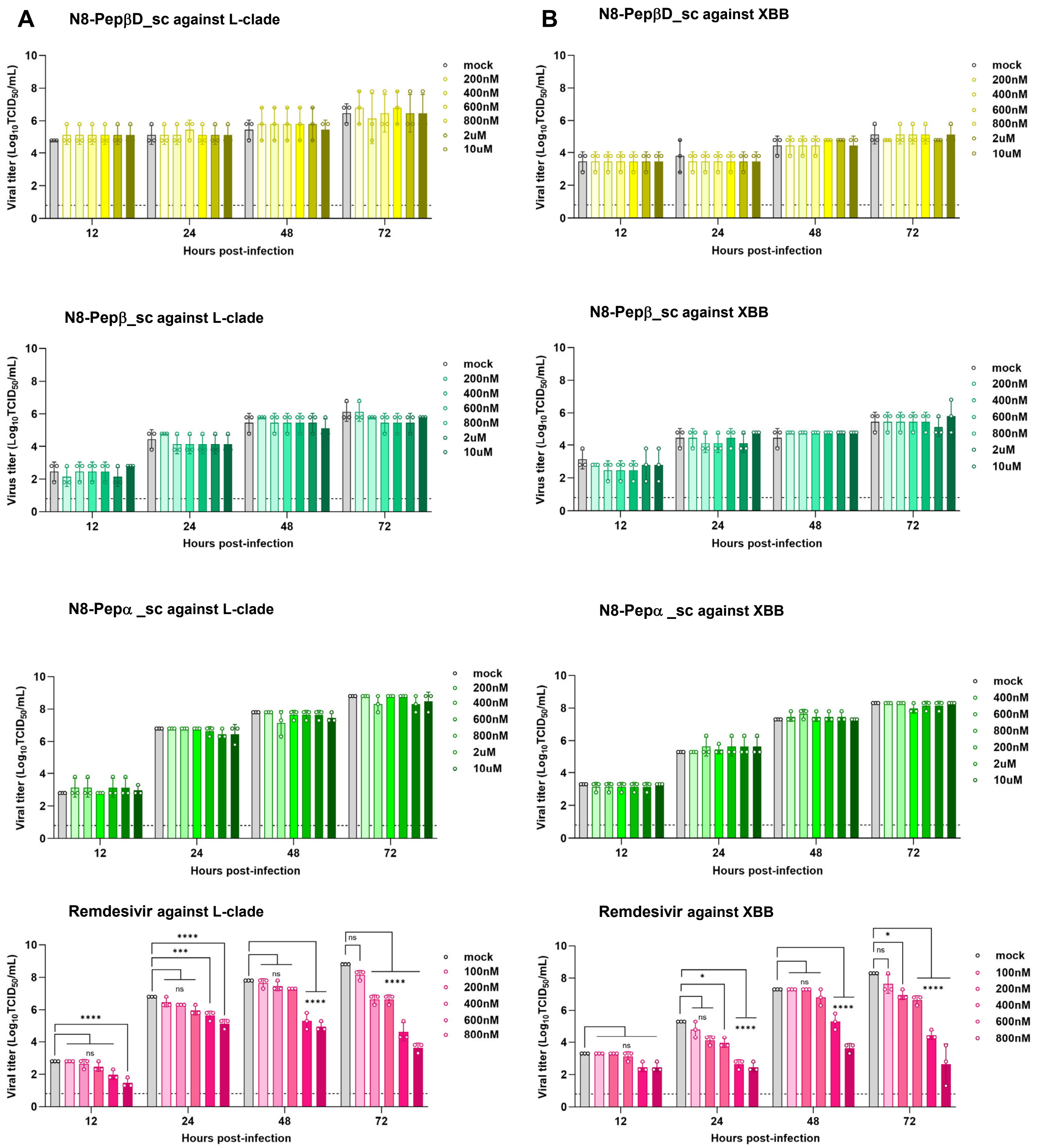

Figure S6. Scrambled NSP8-based peptides and Remdesivir in SARS-CoV-2 replication. SARS-CoV-2 infectious virus titers in Vero E6 cells following different NSP8-based peptide or Remdesivir treatment. Peptides treatment against the (A) L-clade and (B) XBB omicron subvariant, respectively.

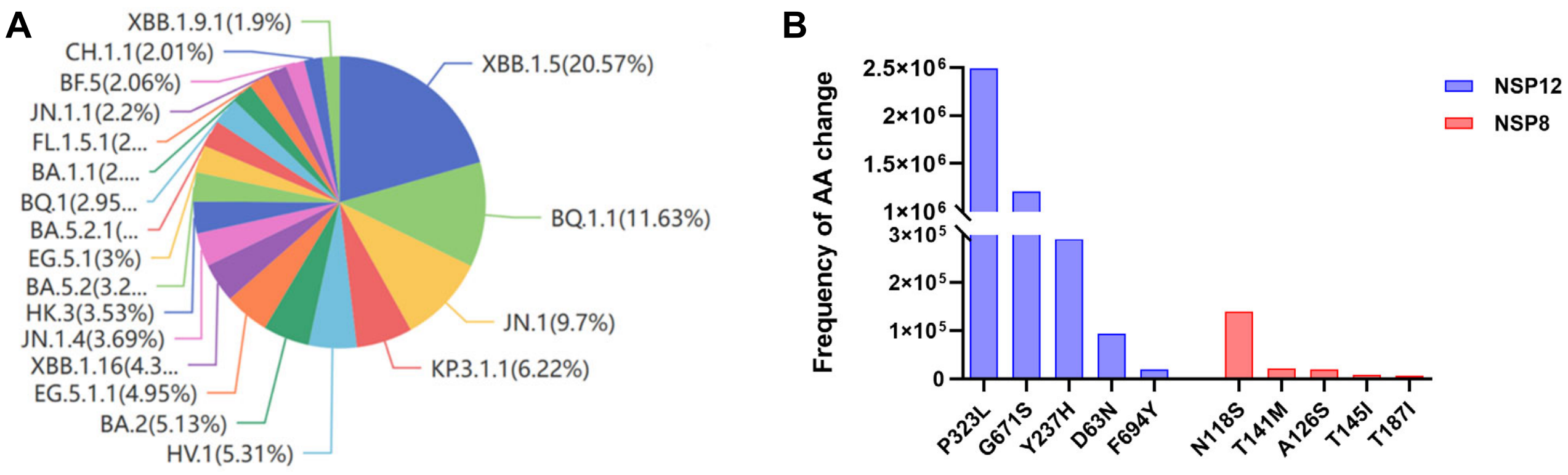

Figure S7. Analysis of NSP12 and NSP8 Mutations in SARS-CoV-2 Lineages
(A) The top 20 SARS-CoV-2 lineages from virus sequences collected between January 1, 2023, and November 18, 2024, were analyzed to assess the frequency of NSP12 and NSP8 mutations. Data and visualization obtained from GISAID's EpiCoV™ database (https://www.gisaid.org). (B) Frequency of amino acid change found in the NSP12 and NSP8.

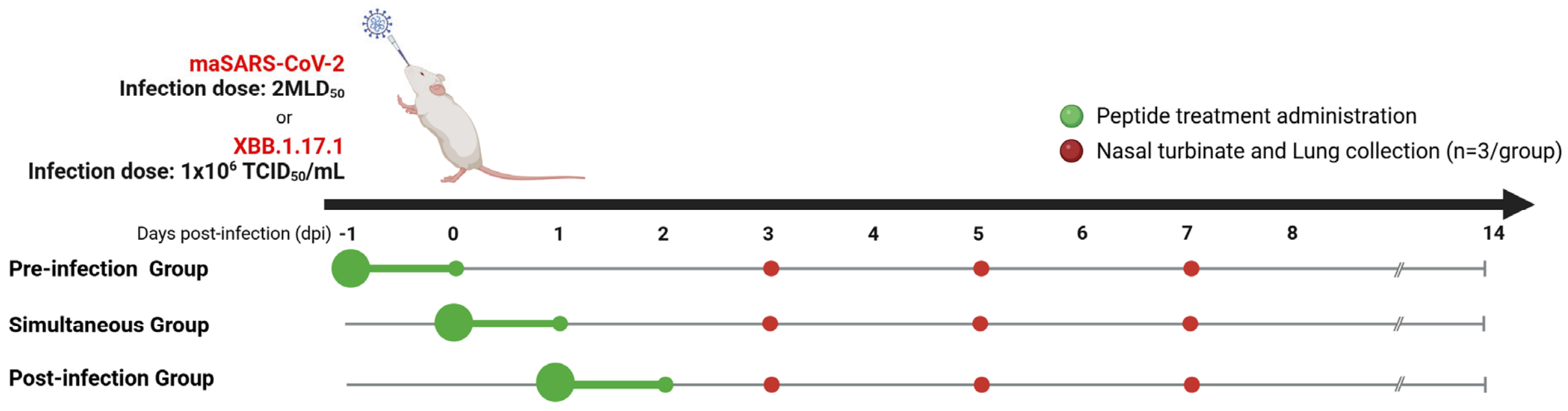

Figure S8. Schematic diagram of experimental design.
Balb/c mice were intranasally inoculated with maSARS-CoV-2 (L clade) at a dose of 2MLD50 or XBB.1.17.1 at a dose of 1×10^6 $TCID_{50}$/mL . Signs of clinical disease were monitored for 14 days post-infection.

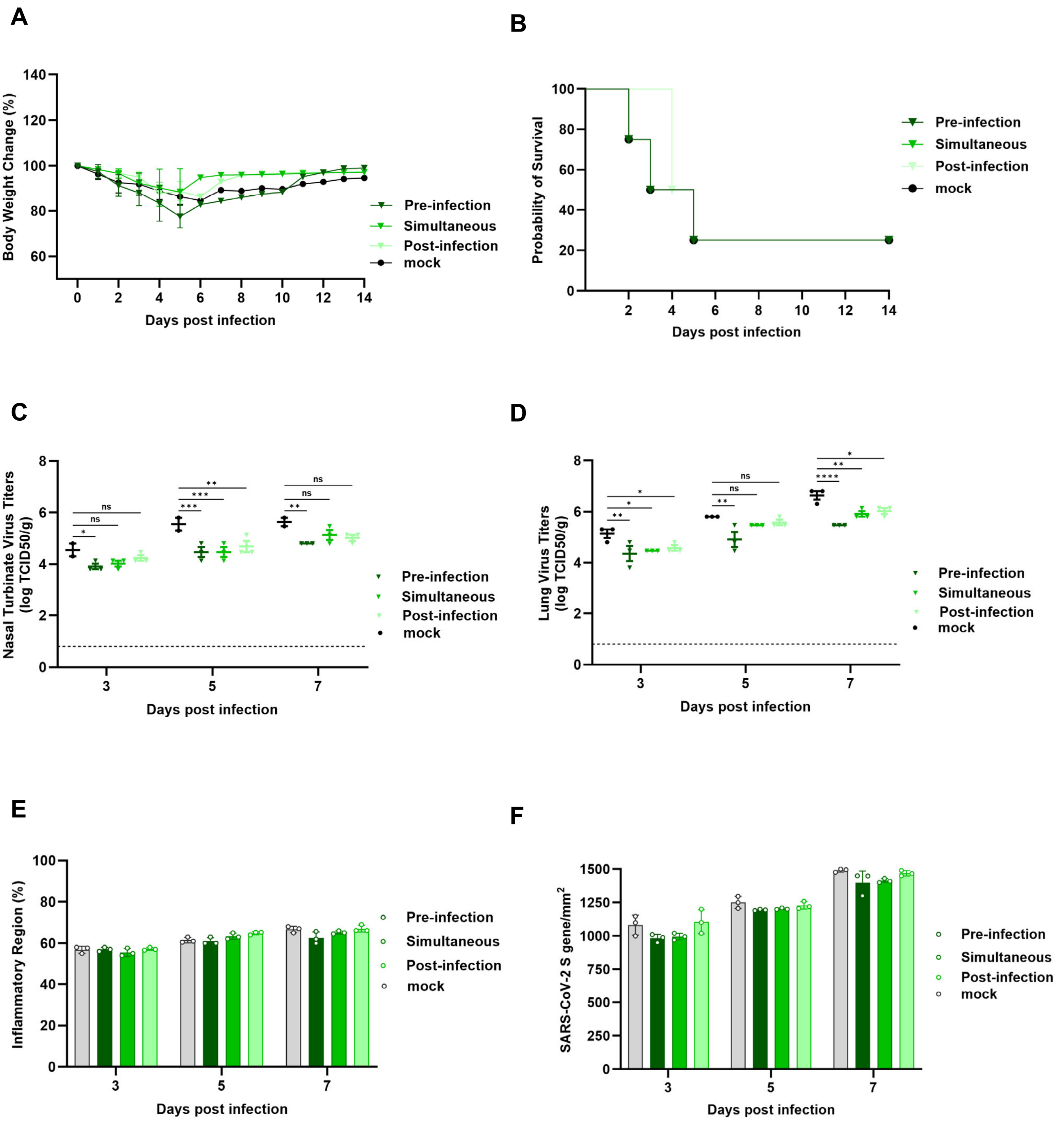

Figure S9. N8-Pepα _sc (Negative Control) Peptide Animal Study data.
N8-Pepβ_sc-treated mice after challenge with maSARS-CoV-2, (A) Body weight and (B) survivability were monitored for 14 days ppost-infection (dpi). (C) Nasal turbinates and (D) Lung viral titers were monitored at 3, 5, and 7 dpi. (E) Inflammatory scoring and (F) presence of replicating SARS-CoV-2 virus (RNAscope) were also evaluated in the lung tissues.

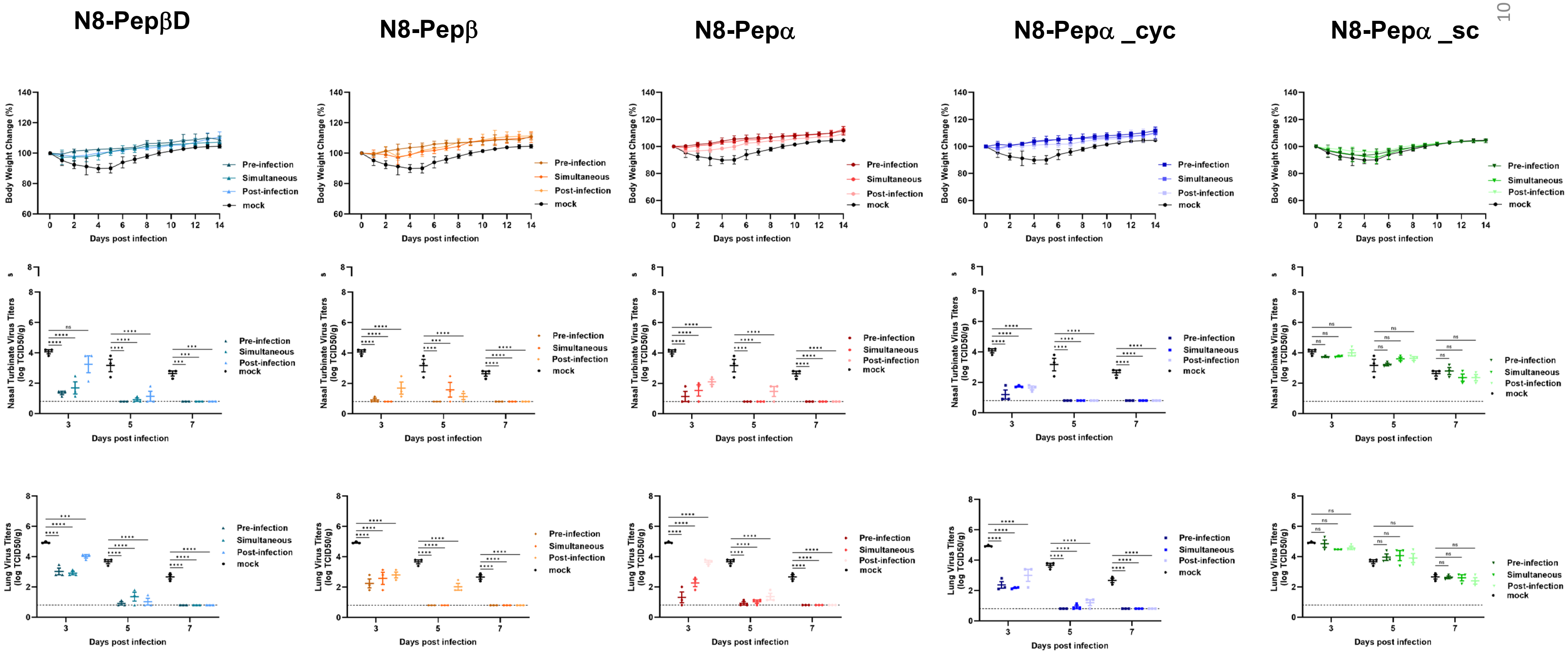

Figure S10. NSP8-Based Peptides Suppress SARS-CoV-2 XBB Replication and Enhance Survival in Infected Mice
Balb/c mice (n=14 per group) were intranasally inoculated with 1×10⁶ $TCID_{50}$/mL of XBB.1.17.1. The treatments—N8-Pepα, N8-Pepα_cyc, N8-Pepα_sc, N8-Pepβ, and N8-PepβD—were administered either prophylactically, concurrently with infection, or post-infection. Clinical signs of infection, body weight changes, and survival rates were monitored over 14 days post-infection (dpi).Groups of mice (n=3 per group) were euthanized at 3, 5, and 7 dpi to measure infectious virus titers in nasal turbinates and lung tissues. Virus titers were quantified as TCID50 in Vero E6 cells, with a dashed line indicating the detection limit (0.8 $TCID_{50}$ /g). The *p* values were calculated using a two-way ANOVA with Dunnett's multiple comparison test. *, *p*<0.5; **, *p*<0.01; *p*<0.001; ***, *p*<0.0001.

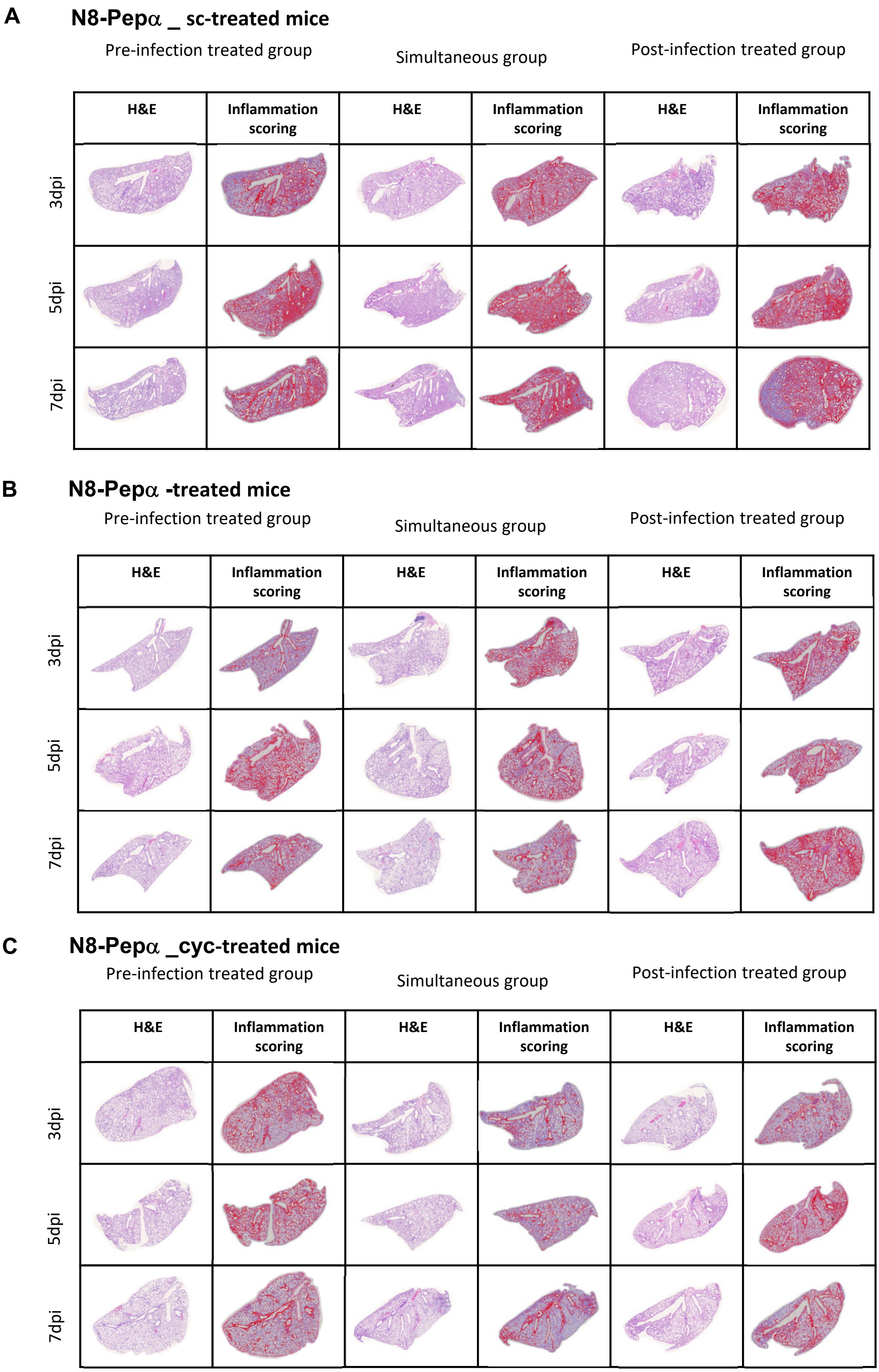

Figure S11. H&E with inflammatory scoring

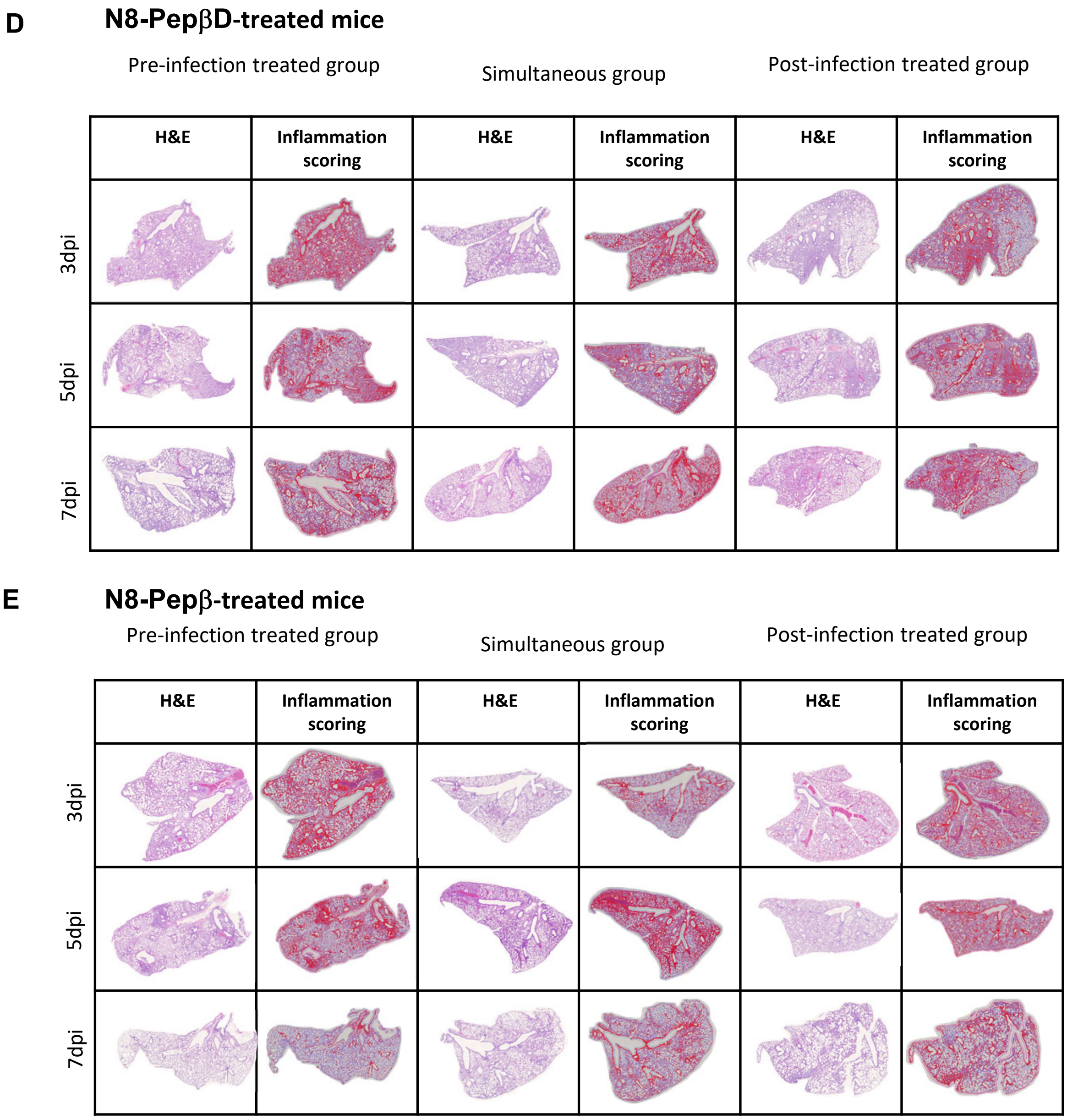

Figure S11. H&E with inflammatory scoring

Representative hematoxylin and eosin (H&E) stained lung tissue sections and corresponding inflammation scoring from mice treated with the NSP8-based peptide; (A) N8-Pepα _sc, (B) N8-Pepα, (C) N8-Pepα _cyc, (D) N8-PepβD, and (E) N8-Pepβ. Tissues were harvested at 3, 5, and 7 days post-infection (dpi) to assess histopathological changes and inflammatory responses. The degree of inflammation is quantified and depicted alongside the corresponding H&E-stained sections

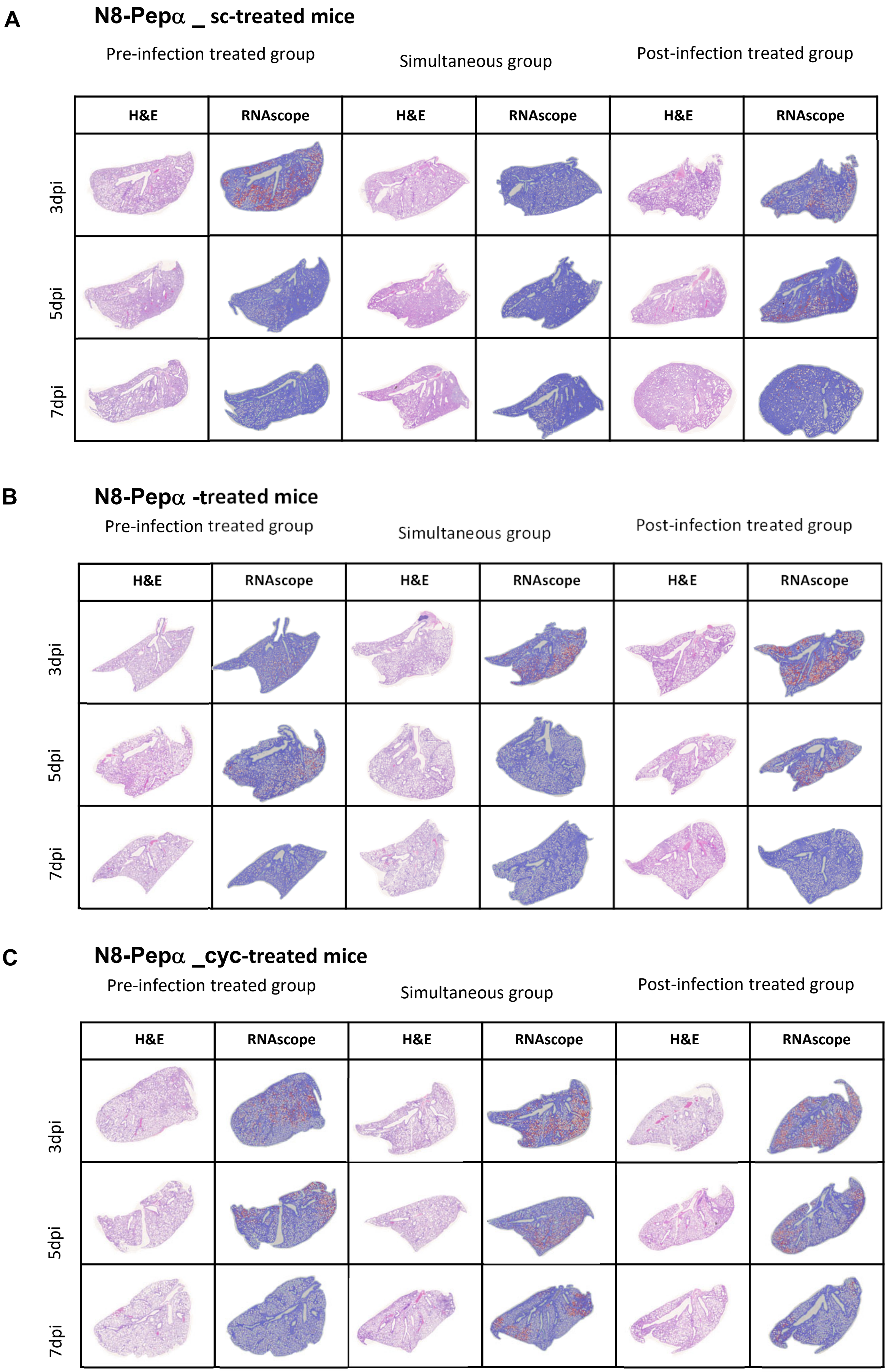

Figure S12 . H&E with RNAscope analysis

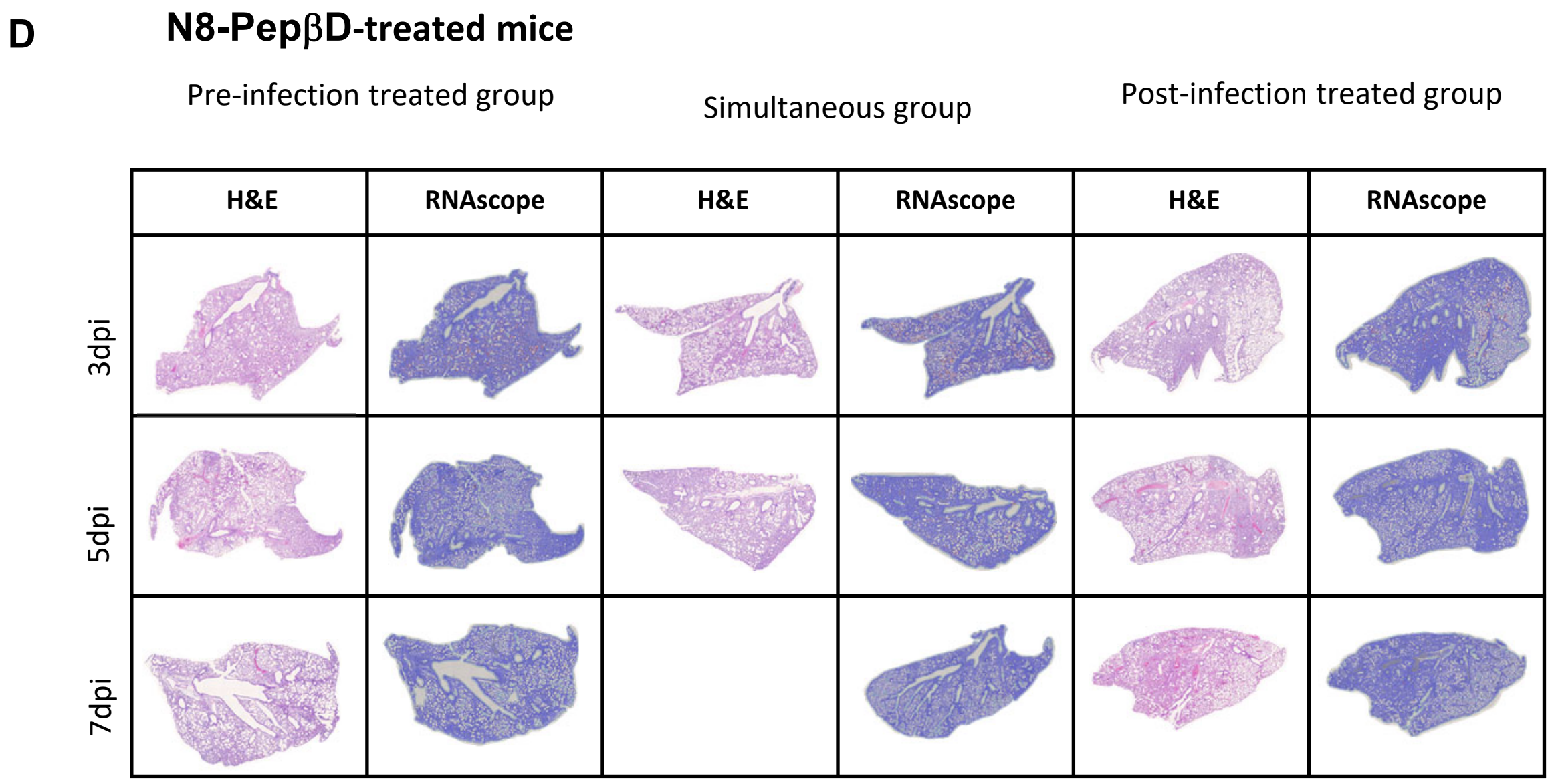

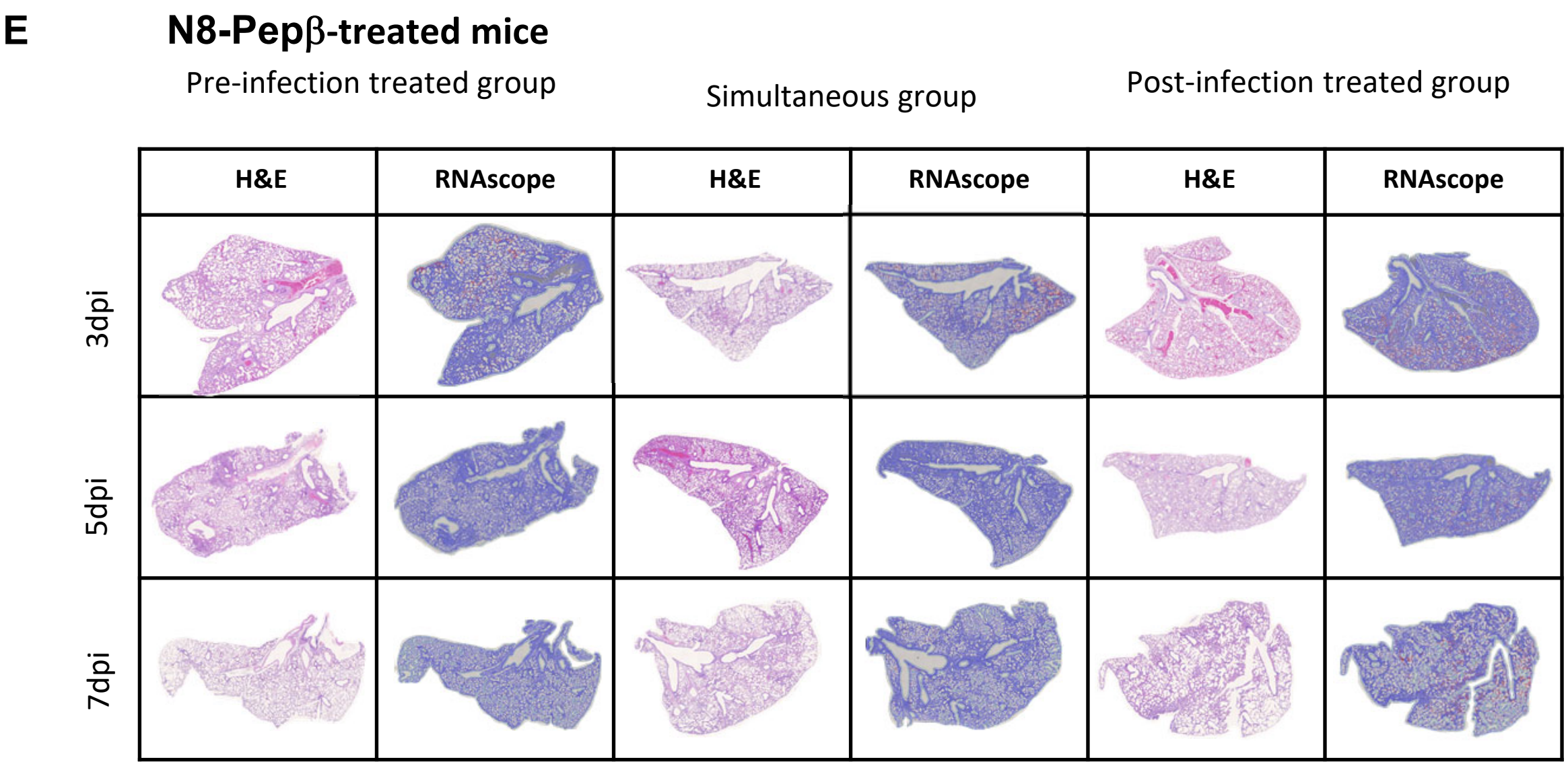

Figure S12. H&E with RNAscope analysis

Representative images of hematoxylin and eosin (H&E)-stained lung tissue sections and RNAscope in situ hybridization were obtained from mice treated with various NSP8-based peptides: (A) N8-Pepα _sc, (B) N8-Pepα, (C) N8-Pepα _cyc, (D) N8-PepβD, and (E) N8-Pepβ. Lung tissues were collected at 3, 5, and 7 days post-infection (dpi) to evaluate the peptides' ability to inhibit SARS-CoV-2 replication. Quantification of positive foci is presented alongside corresponding H&E-stained images.

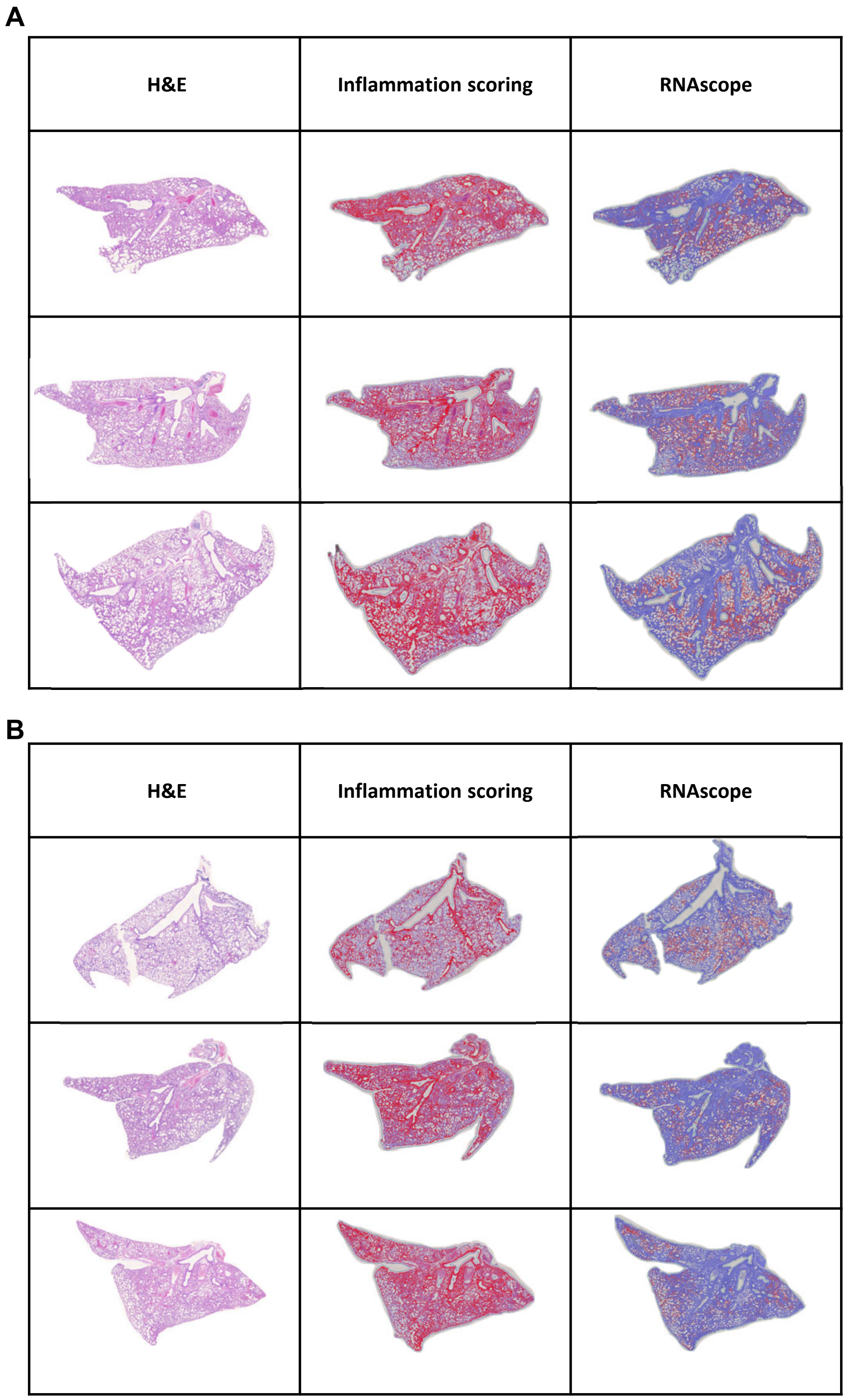

Figure S13. H&E, Inflammation scoring and RNAscope of mock-treated mice (. (A) mock-treatment control for the N8-Pepα, N8-Pepα_cyc experiment, (B) mock-treated control for the N8-Pepβ, and N8-PepβD animal experiment.

Table S1. Statistics for the best refined model from HADDOCK

	N8-Pepα	N8-Pepα_cyc	N8-Pepβ	N8-PepβD
HADDOCK score	-111.9 ± 4.0	-113.2 ± 5.3	-159.3 ± 3.3	-158.6 ± 1.2
Cluster size	7	12	10	26
RMSD from the overall lowest-energy structure	0.5 ± 0.3	0.4 ± 0.2	0.4 ± 0.3	0.3 ± 0.2
Van der Waals energy	-74.2 ± 2.4	-83.2 ± 4.9	-102.1 ± 2.7	-108.9 ± 4.9
Electrostatic energy	-233.8 ± 14.5	-213.5 ± 12.6	-276.0 ± 28.7	-202.6 ± 27.7
Desolvation energy	-10.4 ± 0.6	-9.8 ± 2.6	-13.6 ± 3.1	-17.6 ± 1.9
Restraints violation energy	194.8 ± 27.2	224.8 ± 55.0	117.3 ± 25.4	84.3 ± 21.4
Buried Surface Area	2144.9 ± 25.6	2289.3 ± 24.5	2870.5 ± 31.8	2629.5 ± 56.6
Z-Score	-1.9	-2.6	-2.8	-2.5

Reported values represent mean ± SD from the best-scoring cluster. RMSD is measured from the overall lowest-energy structure. Energies are in kcal/mol; buried surface area (BSA) is in Å². Lower Z-scores indicate higher-quality models.

Table S2: Kinetic Binding Parameters Determined by SPR

Peptides	k_{on} ($M^{-1}s^{-1}$)	k_{off} (s^{-1})	K_D (nM)
N8-Pepα	1.21×10^4	0.53×10^{-3}	42.2 ± 4.3
N8-Pepα_cyc	1.57×10^4	1.04×10^{-3}	66.5 ± 4.8
N8-Pepβ	1.21×10^4	0.52×10^{-3}	43.7 ± 4.8
N8-PepβD	39.6×10^4	0.90×10^{-3}	2.3 ± 0.8

Table S3: Summary of plasma and microsomal stability of NSP8-based Peptides

Peptide	Plasma Stability	Microsomal Stability				Metabolic Stability Evaluation
	Half-life (min)	Half-life (min)	Remaining at 60 min (%)	*CL_int*, microsomal (µL/min/mg)	*CL_int*, in vivo (mL/min/kg)	
N8-Pepα	3.0	9.2	0.0	151.0	594.7	High metabolism
N8-Pepα-cyc	3.0	23.3	14.9	59.6	234.7	High metabolism
N8-Pepβ	3.0	9.8	15.1	142.0	559.2	High metabolism
N8-PepβD	3.0	17.8	7.8	77.9	306.7	High metabolism

Plasma and liver microsomal half-lives ($t_{1/2}$) were determined by LC-MS/MS analysis. Microsomal intrinsic clearance (*CL_int*, microsomal) was calculated based on the depletion rate, and scaled to in vivo liver clearance (*CL_int*, in vivo) using established mouse parameters. All peptides were classified as high-metabolism compounds based on in vivo *CL_int* values.
